# Host–Pathogen Responses to Pandemic Influenza H1N1pdm09 in a Human Respiratory Airway Model

**DOI:** 10.3390/v12060679

**Published:** 2020-06-24

**Authors:** Elizabeth A. Pharo, Sinéad M. Williams, Victoria Boyd, Vinod Sundaramoorthy, Peter A. Durr, Michelle L. Baker

**Affiliations:** 1 Commonwealth Scientific and Industrial Research Organisation (CSIRO) Health and Biosecurity Business Unit, Australian Centre for Disease Preparedness (ACDP), Geelong, VIC 3220, Australia; elizabeth.pharo@csiro.au (E.A.P.); sinead.williams@csiro.au (S.M.W.); vicky.boyd@csiro.au (V.B.); 2 CSIRO Australian Animal Health Laboratory (AAHL) Business Unit, ACDP, Geelong, VIC 3220, Australia; vinod.sundaramoorthy@csiro.au (V.S.); peter.durr@csiro.au (P.A.D.); 3 School of Medicine, Deakin University, Waurn Ponds, VIC 3216, Australia

**Keywords:** epithelium, lung, innate immune system, cytokine, chemokine, antiviral, tight junction, cell death, cytoskeleton, inflammation

## Abstract

The respiratory Influenza A Viruses (IAVs) and emerging zoonotic viruses such as Severe Acute Respiratory Syndrome-Coronavirus-2 (SARS-CoV-2) pose a significant threat to human health. To accelerate our understanding of the host–pathogen response to respiratory viruses, the use of more complex in vitro systems such as normal human bronchial epithelial (NHBE) cell culture models has gained prominence as an alternative to animal models. NHBE cells were differentiated under air-liquid interface (ALI) conditions to form an in vitro pseudostratified epithelium. The responses of well-differentiated (wd) NHBE cells were examined following infection with the 2009 pandemic Influenza A/H1N1pdm09 strain or following challenge with the dsRNA mimic, poly(I:C). At 30 h postinfection with H1N1pdm09, the integrity of the airway epithelium was severely impaired and apical junction complex damage was exhibited by the disassembly of zona occludens-1 (ZO-1) from the cell cytoskeleton. wdNHBE cells produced an innate immune response to IAV-infection with increased transcription of pro- and anti-inflammatory cytokines and chemokines and the antiviral *viperin* but reduced expression of the mucin-encoding *MUC5B*, which may impair mucociliary clearance. Poly(I:C) produced similar responses to IAV, with the exception of *MUC5B* expression which was more than 3-fold higher than for control cells. This study demonstrates that wdNHBE cells are an appropriate ex-vivo model system to investigate the pathogenesis of respiratory viruses.

## 1. Introduction

Over the past 30 years, there has been a rapid rise in emerging infectious diseases both of zoonotic and human origin [1]. The recent emergence and worldwide spread of the Severe Acute Respiratory Syndrome-Coronavirus-2 (SARS-CoV-2) virus which produces the Coronavirus disease 2019 (COVID-19) respiratory illness demonstrates the risk to human health and the economy posed by respiratory pathogens [2]. For novel viruses, often no diagnostics or therapeutics exist, hence they present an enormous risk of a catastrophic global pandemic [3,4]. Respiratory RNA viruses, particularly influenza A viruses (IAV) of the *Orthomyxoviridae* family are a major pandemic risk as they replicate rapidly, lack a proof-reading mechanism, have a high mutation rate and are readily transmissible [3,5,6,7]. Over 100 years ago, the 1918 H1N1 “Spanish flu” pandemic killed up to 50 million people worldwide [8,9,10]. Three global influenza pandemics have occurred since, “Asian flu” H2N2 in 1957, “Hong Kong flu” H3N2 in 1968 and “swine flu” in 2009 (A/H1N1pdm09) which emerged in Mexico [11,12,13,14] and is still circulating as a seasonal IAV strain [15,16,17]. These outbreaks killed an estimated range of between 2.1 and 8.4 million people [18]. Highly pathogenic pandemic IAV strains, plus the avian H5N1 can cause massive viral pneumonia, multiple organ failure and death, due to hyperinflammation caused by the excessive production of cytokines and chemokines, or “cytokine storm” [19,20,21,22]. However, each year there are 1 billion cases of seasonal influenza worldwide and up to 645,000 deaths [23].

The airway epithelium is the initial site of influenza virus infection [24]. Hence, it must provide the first line of host defense, forming a physicochemical barrier against inhaled toxins, pathogens, allergens and particulates, preventing their entry into the bloodstream [25,26,27]. It also plays an essential role in the mucociliary clearance of foreign matter [28,29] and regulates innate and adaptive immune system responses, prevents inflammation and is involved in tissue remodeling and repair [27,30,31,32]. The structural integrity of the airway epithelium is provided by apical junction complexes (AJCs) which form highly regulated and selectively permeable barriers between adjacent cells [27,33]. IAV and other pathogens can damage the epithelium by disrupting AJCs [34,35]. Therefore, AJC damage is a key diagnostic of lung disease [34,35]. AJCs are complex structures that include tight junctions (TJs), adherens junctions (AJs) and desmosomes, while gap junctions also provide cell–cell connectivity [27,31,36,37,38]. Peripheral intracellular scaffolding proteins are also essential for TJ assembly as they form bridges between transmembrane junctional proteins and the filamentous cytoskeleton [12,39,40,41]. Tight junction protein 1, also known as zonula occludens (ZO)-1, forms a link between TJs, the cytoskeleton and AJs and hence plays a vital role in AJC stability [32,38,39,40,42,43].

In the past few decades, there has been a concerted effort to develop physiologically relevant human lung cell culture models to study respiratory viruses and develop new therapeutics [44,45,46,47]. Well-differentiated, primary normal human bronchial epithelial (wdNHBE) cells, also known as human airway epithelial (HAE) cells, grown at the air-liquid interface (ALI, or “airlifted”) reproduce the in vivo airway epithelium in vitro [25,48]. They form AJCs, exhibit ion transport, mucus secretion and mucociliary clearance [25,48,49,50,51]. wdNHBE cells also produce host–pathogen responses when exposed to respiratory viruses, such as the production of antiviral, immune and pro- and anti-inflammatory molecules [52,53], as occurs in the host in vivo (reviewed in: [20,21,22,27,54,55,56,57]). The response of airway epithelial cells (from different donors, cell preparations, etc.) grown at ALI to respiratory viruses has been studied for selected IAV subtypes [53,58,59,60,61,62], Respiratory Syncytial Virus (RSV) [63,64], Rhinovirus [65], Middle East Respiratory Syndrome-Coronavirus (MERS-CoV) [66] and SARS-CoV [67,68,69] and the polyinosinic-polycytidylic acid [poly(I:C)] dsRNA viral mimic [70,71].

The major aims of our study were to establish a human airway model that could be used for future investigation of emerging respiratory viruses and the testing of therapeutics. Commercially sourced wdNHBE cells grown at ALI on transwells consistently reproduced the pseudostratified airway epithelium in vitro and were challenged with pandemic IAV H1N1pdm09 and the poly(I:C) dsRNA viral mimic. Poly(I:C) was chosen as a positive control for the stimulation of the innate immune response. Significant differences in the response of the in vitro bronchial epithelium to these challenges were identified which provide new insights into the pathogenesis of pandemic influenza H1N1pdm09. To our knowledge, this is the first study to demonstrate that an IAV strain dissociates ZO-1 from apical junction complexes and the cell cytoskeleton. Furthermore, the downregulation of *MUC5B* in H1N1pdm09-infected cells suggests that mucociliary clearance of the virus from the airways may be impaired thereby contributing to pathological effects of the virus. Our data provides new insights into the host–pathogen response in airlifted NHBE cells for multiple immune system challenges. 

## 2. Materials and Methods 

### 2.1. Cell Culture

Normal human bronchial epithelial cells from a healthy, nonsmoking donor (lot#: 0000485960; Lonza, Walkersville, MD, USA) were well-differentiated, i.e., grown at ALI [25,48], with several modifications. NHBE cells were thawed, seeded at 5000 cells/cm^2^ and expanded in Bronchial Epithelial Growth Medium (BEGM) at 37 °C/5% CO_2_ as described by the manufacturer (Lonza, Walkersville, USA). However, all cells were cultured without antibiotics. Cells were passaged when they were 65–70% confluent. Briefly, cells were rinsed with Dulbecco’s phosphate buffered saline (DPBS), trypsinized for 5–7 min with Animal Component Free (ACF) Enzymatic Dissociation Solution, the enzyme inactivated with ACF Enzyme Inhibition Solution (STEMCELL Technologies, Vancouver, BC, Canada), the cells mixed gently and centrifuged at 300× *g* for 5 min, counted and passage 2 stocks for experiments frozen down (1 × 10^5^ cells/mL). 

Passage 2 cells were thawed, seeded at 5000 cells/cm^2^ and expanded in PneumaCult-Ex Plus Medium (STEMCELL Technologies, Canada). Cells were passaged at 65–70% confluence as previously described and undifferentiated NHBE cells seeded at 2.1 × 10^5^ cells/cm^2^ on the apical surface of a 6.5 mm diameter transwell polyester membrane (0.33 cm^2^; 0.4 µm pores; Corning, Corning, NY, USA) that had been precoated with Gibco Collagen I Rat Protein, Tail (5 µg/cm^2^; (ThermoFisher Scientific, Waltham, MA, USA). Cells were grown in PneumaCult-Ex Plus (STEMCELL Technologies, Canada) under liquid covered culture conditions with media in both the apical and basolateral compartments. Once confluent, approximately 3–4 days postseeding, cells were cultured at ALI with PneumaCult-ALI Maintenance Media (STEMCELL Technologies, Canada; hereafter referred to as ALI media) in the basolateral compartment only and media changed every 2–3 days. Around 4 weeks postairlift, well-differentiated NHBE (wdNHBE) cells form a polarized pseudostratified epithelium of basal, goblet, club and ciliated cells, with cells subsequently used for experiments 4–8 weeks postairlift. 

Madin Darby canine kidney (MDCK; ATCC CCL-34, ATCC Gaithersburg, MD, USA) cells were cultured in Eagle’s Minimal Essential Medium (EMEM) with media supplemented to a final concentration of 10% (v/v) fetal calf serum (FCS), 10 mM HEPES and 100 µg/mL penicillin/streptomycin (ThermoFisher Scientific, USA). Cell cultures were maintained at 37 °C with 5% CO_2_. 

### 2.2. Histology and Immunocytochemistry

Undifferentiated NHBE and wdNHBE cells were fixed in 10% neutral buffered formalin and embedded in paraffin, sectioned (5 µm; microtome), mounted and stained with hematoxylin and eosin. For immunofluorescence assays, airlifted NHBE cells were fixed in 4% *w/v* paraformaldehyde for at least 20 min, rinsed with and then stored in DPBS at 4 °C. Fixed cells were permeabilized for 15 min at RT with 1% *v/v* Triton X-100/DPBS (Sigma-Aldrich, St. Louis, MO, USA), blocked for 1 h in 1% *w/v* bovine serum albumin (BSA; Sigma-Aldrich, St. Louis, MO, USA)/0.5% *v/v* Triton X-100/DPBS. Apoptotic DNA fragmentation which occurs in the last phase of apoptosis (programmed cell death) was detected by Terminal deoxynucleotidyl transferase dUTP nick end labelling (TUNEL) staining using the In Situ Cell Death Detection Kit, TMR red (Cat# 12 156 792 910; Roche, Mannheim, Germany) according to the manufacturer’s instructions. Cells were then stained with primary antibody overnight at 4 °C, washed three times with DPBS and then incubated with the appropriate species-specific secondary antibody diluted at 1:200 in 1% *w/v* BSA/0.5 % *v/v* Triton X-100/DPBS for at least 1 h at RT. Transwell membranes were rinsed three times with DPBS, stained with phalloidin (A22287, Alexa Fluor 647 phalloidin, ThermoFisher Scientific, USA) which stains F-actin filaments for 20 min at RT, washed three times with DPBS, stained with the nuclear stain 4’,6-diamidino-2-phenylindole (DAPI, D1306; ThermoFisher Scientific, USA) for 10 min at RT. Transwells were washed twice with sterile water, excised and mounted on glass slides with ProLong Gold Antifade Mountant (P10144, ThermoFisher Scientific, USA).

Primary antibodies were used at the following dilutions 1:50 mucin 5AC (MUC5AC, MA5-12178, ThermoFisher Scientific, USA), 1:200 mucin 5B (MUC5B, HPA008246, Sigma-Aldrich, USA), 1:50 ZO-1 (ZO-1-1A12; 33-9100, ThermoFisher Scientific, USA), 1:100 acetylated tubulin (TubAc, T7451, Sigma-Aldrich, USA) and 1:100 claudin 4 (CLDN4, ab53156, Abcam, Cambridge, UK). A monoclonal antibody specific to the nucleoprotein (NP) of type A specific Influenza (IAV NP) was produced using hybridoma technology in mice, as previously described [72]. The hybridoma IAV NP monoclonal antibody H16-L10-4 used at 1:50 dilution was kindly provided by Paul Selleck (Australian Centre for Disease Preparedness, CSIRO, Geelong, Australia). Secondary antibodies were purchased from ThermoFisher Scientific, USA and included goat anti-Mouse IgG (H+L), Alexa Fluor 488 and Alexa Fluor 568 (A11029 and A11031 respectively), goat anti-rabbit IgG (H+L), Alexa Fluor 488 (A11034) and donkey anti-rabbit IgG (H+L) Alexa Fluor 568 (A10042).

### 2.3. Confocal Imaging

Cells were imaged using the Zeiss LSM 800 confocal microscope (ZEISS, Oberkochen, BW, Germany) using a 40× oil immersion objective unless specified otherwise. Images were captured as Z-stacks and maximum intensity projections generated. Images were captured and processed using ZEN 2.5 Blue software (ZEISS, Germany).

### 2.4. Fluorescence Activated Cell Sorting

The presence of α2-3- and α2-6-linked sialic acids (SAs) on the surface of wdNHBE cells was analyzed by fluorescence activated cell sorting (FACS). wdNHBE cells were detached from transwell membranes by two 30 min incubations with prewarmed (37 °C) Versene Solution (ThermoFisher Scientific, USA). Cells were resuspended and incubated with biotinylated *Sambucus nigra* agglutinin I (SNA I, B-1305, Vector Laboratories, Burlingame, CA, USA) or *Maackia amurensis* agglutinin II (MAA II) lectins (B-1265, Vector Laboratories, USA) in FACS buffer (0.5% BSA in DPBS). Cells were blocked with FACS buffer, incubated with DyLight 488 Streptavidin (SA-5488, Vector Laboratories, USA) and washed with DPBS. Cells were sorted with the Becton Dickson (BD) FACSAria II cytometer and analyzed using FACSDiva Version 6.1.2 software (BD, Franklin Lakes, NJ, USA).

### 2.5. Virus Propagation

The 2009 pandemic strain of influenza virus A/California/7/2009 (H1N1)pdm09, kindly provided by the WHO Collaborating Centre for Reference and Research, Melbourne, VIC, Australia was propagated by allantoic cavity inoculation of 10 day old embryonated chicken eggs at 37 °C for 48 h. The virus was passaged once in MDCK cells in the presence of 2 µg/mL L-1-Tosylamide-2-phenylethyl chloromethyl ketone (TPCK)-treated trypsin (Sigma-Aldrich, USA) at 37 °C for 72 h or until cytopathic effect was observed. Virus was aliquoted and titrated to determine the median tissue culture infectious dose (TCID_50_) on MDCK cells in the presence of 2 µg/mL TPCK-treated trypsin. Briefly, virus was serially diluted 10-fold and applied in quadruplicate to cell monolayers. Cytopathic effect was assessed five days post infection. Viral titres were calculated according to the method of Reed–Muench as TCID_50_/mL [73].

### 2.6. Immune Challenge of wdNHBE Cells with Influenza a Virus and Poly (I:C)

wdNHBE cells were washed three times with ALI media to remove excess mucus from the upper surface and inoculated with Influenza A/H1N1pdm09 (100 µL) at a multiplicity of infection (MOI) of 1.0 for 1 h at 37 °C. The virus inoculum was removed, the cells washed three times with ALI media to remove unbound virus and the cells incubated at 37 °C for 30 h postinfection (experiment endpoint). At each time point (6, 18, 24 and 30 h postinfection), the virus was harvested by the addition of ALI media to the upper surface for 30 min at 37 °C, with both apical and basolateral media collected and stored at −80 °C for endpoint analyses.

In addition to IAV challenge, wdNHBE cells were inoculated with varying concentrations of poly(I:C) dsRNA viral mimic [Poly(I:C) High Molecular Weight, Cat: tlrl-pic, InvivoGen, San Diego, CA, USA]. Briefly, airlifted cells were washed in prewarmed DPBS for 15 min at 37 °C to remove excess mucus. Poly(I:C) (20 µg and 30 µg) in ALI media was added to the apical surface of wdNHBE cells and ALI media added to the basolateral compartment. Cells were incubated for 24 h at 37 °C, then washed three times with ALI media to remove excess poly (I:C). Basolateral media was replaced and the cells incubated for an additional 48 h at 37 °C.

### 2.7. Transepithelial Electrical Resistance

Transepithelial electrical resistance (TEER) across the epithelium of undifferentiated and wdNHBE cells was measured using an epithelial voltohmmeter 2 (EVOM2) with an STX2 chopstick electrode (World Precision Instruments, Sarasota, FL, USA) [74]. ALI media was added to the apical and basolateral compartments, equilibrated for at least 15–20 min at RT and the TEER measured. TEER readings were membrane-corrected by subtraction of measurements from control transwells (no cells) and expressed in units of Ω or Ω × cm^2^.

### 2.8. Paracellular Permeability

Dextran conjugated to fluorescein isothiocyanate (FITC-dextran, 4 kDa; Sigma-Aldrich, USA) was used to determine the paracellular permeability of wdNHBE cells, i.e., the transport of FITC-dextran from the apical to the basolateral compartment of the transwell via extracellular space in the pseudostratified airway epithelium [74]. FITC-dextran diluted in ALI media (0.5 mg/mL) was added to the upper transwell compartment and ALI media alone added to the basolateral compartment and the cells incubated for 3 h at 37 °C. Basolateral media was mixed, sampled in triplicate in a microtiter plate and FITC-dextran analyzed on a BioTek Synergy HT microtitre plate reader. Assay standards and sample solutions were prepared concurrently.

### 2.9. Lactate Dehydrogenase Release Assay

Lactate dehydrogenase (LDH) release was quantified using the CytoTox 96 Non-Radioactive Cytotoxicity assay (G1780, Promega Corporation, Madison, WI, USA) according to the manufacturer’s instructions. Controls including no cells, untreated cells and maximum LDH release (Triton X-100-treated cells, 100% cytotoxicity) were analyzed together with the apical samples using a BioTek Synergy HT microtitre plate reader. Cytotoxicity percentage was expressed as experimental LDH release compared to maximum LDH release for Triton X-100 treated cells.

### 2.10. Total RNA Extraction, cDNA Synthesis, Quantitative Reverse Transcription PCR

Quantitative reverse transcription PCR (RT-qPCR) was performed on total RNA extracted from cells. Total RNA was isolated from wdNHBE cells using the RNeasy Mini Kit (Qiagen, Hilden, NW, Germany) with on-column DNase I digestion. Total RNA was quantified using the DS-11 FX spectrophotometer (DeNovix, Wilmington, DE, USA) and first strand cDNA made using the SuperScript III First-Strand Synthesis System (ThermoFisher Scientific, USA), total RNA (500 ng) and oligo(dT)_20_. Gene expression was determined by RT-qPCR using first strand cDNA (1:100 dilution), TaqMan Gene expression Master Mix and TaqMan inventoried probes (Applied Biosystems, Foster City, CA, USA), all of which were exon-spanning apart from the single exon *interferon beta 1* (*IFNB1*) probe (Table S1). RT-qPCR reactions for the IAV H1N1pdm09 and poly(I:C) experiments were performed on the QuantStudio 3 System and QuantStudio 6 Flex Real-Time PCR System (Applied Biosystems, USA) respectively with the appropriate no template controls included on each plate. Samples were assayed in triplicate. Conditions used were 50 °C for 2 min, denaturation at 95 °C for 10 min followed by 40 cycles of 95 °C for 15 s, 60 °C for 1 min. A cycle threshold (CT) of 0.1 was applied to all gene probes. Gene expression levels were normalized to the human *glyceraldehyde 3-phosphate dehydrogenase (GAPDH*) housekeeping gene and expressed as fold over detectable (FoD), as described [75]. Minimum detectable CT was set at 40 cycles.

### 2.11. Bioinformatics Analysis

To assess the effect of IAV H1N1pdm09 infection in wdNHBE cells on the transcription of twelve innate immune genes and two mucin-encoding genes quantified by RT-qPCR, we constructed a gene coexpression network [76]. We used the normalized gene copy number in *n* = 12 transwells at 30 h postinfection with IAV H1N1pdm09, i.e., the number of gene copies per 1000 copies of *GAPDH* (see above) to produce the network. For each pair of genes, the Pearson correlation coefficient (PCC) was calculated to estimate the strength of the linear relationship between gene expression levels. A PCC value exceeding ±0.80 was used as the coexpression threshold. For each gene-pair, a network was constructed with the expressed genes forming the network’s nodes and the PCC values exceeding the 0.80 threshold forming an edge. The PCC values were estimated using the “*corr*” function in the *R stats* package, and the network was constructed and visualized using the *igraph* package [77].

### 2.12. Multiplex Immunoassays

Cytokines and chemokines secreted by wdNHBE cells into the apical and basolateral compartments of transwells were measured in media harvested at 30 h post-H1N1pdm09 infection and at 48 h post-poly(I:C) treatment. Samples were analyzed using multiplex immunoassay kits (High sensitivity 5-Plex: CSF2 (GM-CSF), IL-1β, IL-6, CXCL8 (IL-8), TNF-α Human ProcartaPlex Panel; PPXS-05-MXH497Z; eBioscience, ThermoFisher Scientific, USA). Briefly, ALI media was added to the apical compartment and incubated for 15 min at 37 °C, collected and stored at −20 °C. Samples and ProcartaPlex standards were gamma-irradiated (25 kGy; Steritech, Dandenong South, VIC, Australia), immunoassays performed according to the manufacturer’s instructions and samples analyzed with the Bio-Plex Protein Array System (Bio-Rad Laboratories, Inc., Hercules, CA, USA) set at low RP1 target settings. Binding results represent the average of the median fluorescence intensity (MFI) of at least 100 microspheres per sample.

### 2.13. Statistical Analyses

Statistical analyses for most experiments were performed in GraphPad Prizm 8.2.1 (GraphPad Software, Inc., CA, USA). Data from three independent experiments for each of the IAV and poly(I:C) challenges was pooled and analyzed as described below. For IAV H1N1pdm09 experiments, growth kinetics of IAV H1N1pdm09 (TCID_50_/mL) were analyzed by one-way ANOVA using Tukey’s multiple comparison test; percentage of preinfection TEER, FITC-dextran transported across the epithelium and percentage cytotoxicity were analyzed by multiple t-tests using the two-stage linear step-up procedure of Benjamini, Krieger and Yekutieli, with a False discovery Rate (FDR) of 1%. TEER resistance in IAV H1N1pdm09-inoculated and mock-inoculated wells at 30 h postinfection was also analyzed with R using a mixed effects model using the “*aov*” and the “*lme*” functions in the *R stats* and *nlme* packages respectively [78]. Poly(I:C) TEERs were analyzed by one-way ANOVA with Brown-Forsythe and Welch ANOVA tests and Dunnett’s T3 multiple comparison test. Poly(I:C) FITC-dextran transported and cell cytotoxicity were analyzed by one-way ANOVA and Tukey’s multiple comparisons test. IAV *MUC5AC* and *MUC5B* gene expression was analyzed by unpaired t-tests and for poly(I:C), by one-way ANOVA using Tukey’s multiple comparison test. Cytokines secreted apically form IAV-infected cells were analyzed by unpaired t-tests. 

### 2.14. Ethics Statement

The use of human cells in all experiments was approved by the CSIRO Health and Medical Human Research Ethics Committee (ethics approval #2019_26_LR, 14th May, 2019).

## 3. Results

### 3.1. Characterization of the wdNHBE Air-Liquid Interface (ALI) Cell Culture Model

**Figure 1:**
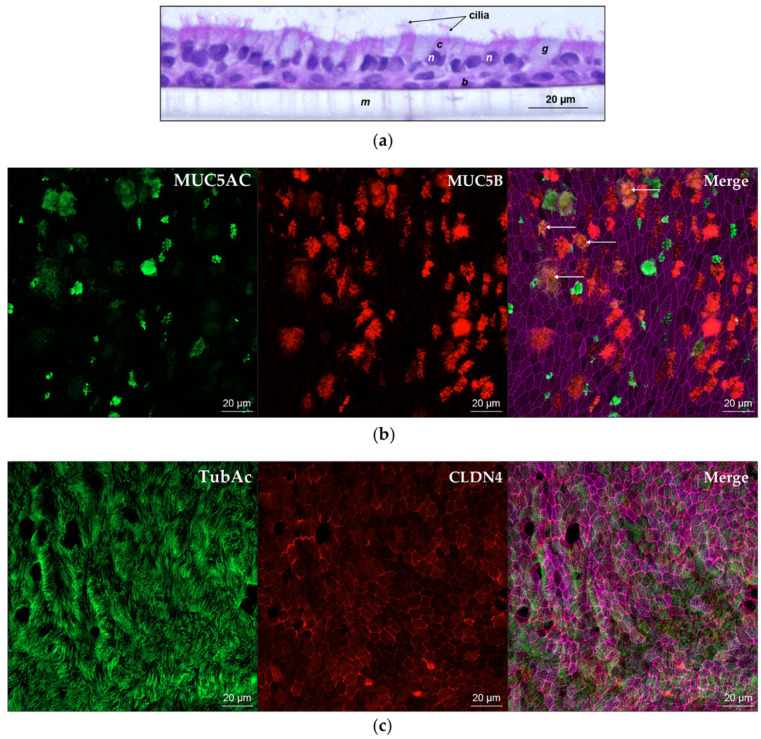
Characterization of wdNHBE cells by histology and immunocytochemistry. (**a**) Hematoxylin and eosin staining of a transverse section of NHBE cells differentiated at the air liquid interface on the apical surface of a transwell membrane. Nuclei (*n*) are stained purple and membranes, cytoplasm and cilia are stained pink. Basal cells (*b*), ciliated cells (*c*) with surface cilia, goblet cells (*g*) and the porous polyester transwell membrane (*m*). (**b**) *En face* images of wdNHBE cells stained with MUC5AC (green, goblet cells) and MUC5B (red, secretory cells). The Merge of MUC5AC, MUC5B and phalloidin (F-actin)-stained cytoskeleton (pink) depicts the partial colocalization of MUC5AC and MUC5B secretion (yellow, indicated by arrows). *En face* Z-stacks of 19 slices (9 µm) were captured using a 40x oil immersion objective and maximum intensity projections generated, 20 µm scale bars indicate cell size. (**c**) Confocal microscopy of wdNHBE cells stained with acetylated tubulin (TubAc, green, cilia on the surface of ciliated cells) and CLDN4 (red, tight junctions). Merge of TubAc, CLDN4 and F-actin. Maximum intensity projections of *en face* Z-stacks, 19 slices (9 µm) imaged using a 40x oil immersion objective with 20 µm scale bars are shown.

Prior to innate immune system challenge studies, we characterized our commercially sourced NHBE cells. Cells cultured at ALI on collagen I-coated transwells formed a polarized, pseudostratified epithelium around four weeks postairlift. wdNHBE cells exhibited a mucociliary phenotype, characterized by the secretion of mucus on the apical surface and the coordinated beating of cilia on the surface of ciliated cells (Video S1). Histological staining confirmed that wdNHBE cells formed a columnar epithelium 2–3 cells thick, consisting of basal, goblet, club and ciliated cells, representative of the human airway epithelium in vivo (Figure 1a). Immunofluorescence assays confirmed the expression of MUC5AC, a goblet cell marker and MUC5B, produced in secretory cells and the partial colocalization of MUC5AC and MUC5B (Figure 1b). Immunostaining of wdNHBE cells also confirmed the presence of ciliated cells, characterized by the expression of acetylated tubulin on cell-surface cilia (Figure 1c). In order to confirm that airlifted cells formed an intact epithelial barrier, we measured the TEER of undifferentiated and differentiated NHBE cells. The TEER indicates the degree of polarization and differentiation of NHBE cells and hence the formation of an intact airway epithelial barrier [74,79]. For undifferentiated NHBE cells, the average TEER was <130 Ω × cm^2^. In contrast, TEER values ≥350 Ω × cm^2^ were recorded for a majority of wdNHBE cells with average values consistently ≥400–500 Ω × cm^2^ (Figure S1; Table S2; Table S3). Therefore, our cells provide an excellent model to study the integrity of the airway epithelium in vitro before, during and after immune system challenges. 

### 3.2. wdNHBE Cells Are Susceptible to Influenza Virus Infection

IAV infects cells by binding to cell-surface sialic acid receptors. Therefore, we characterized the IAV receptors present on the exterior of wdNHBE cells by FACS using the lectins *SNA* I and *MAA* II which bind to α2-6-linked sialic acids (SA) and α2-3-linked SAs respectively. While hemagglutinins of human IAVs preferentially bind α2-6-SA, those from avian IAVs recognize α2-3 SA [61,80]. Almost all (99.2%) wdNHBE cells had α2-6-linked sialic acids on the cell surface, while 73.6% had cell-surface α2-3-linked sialic acids (Figure S2), demonstrating the susceptibility of wdNHBE cells to IAV infection in this study. 

**Figure 2:**
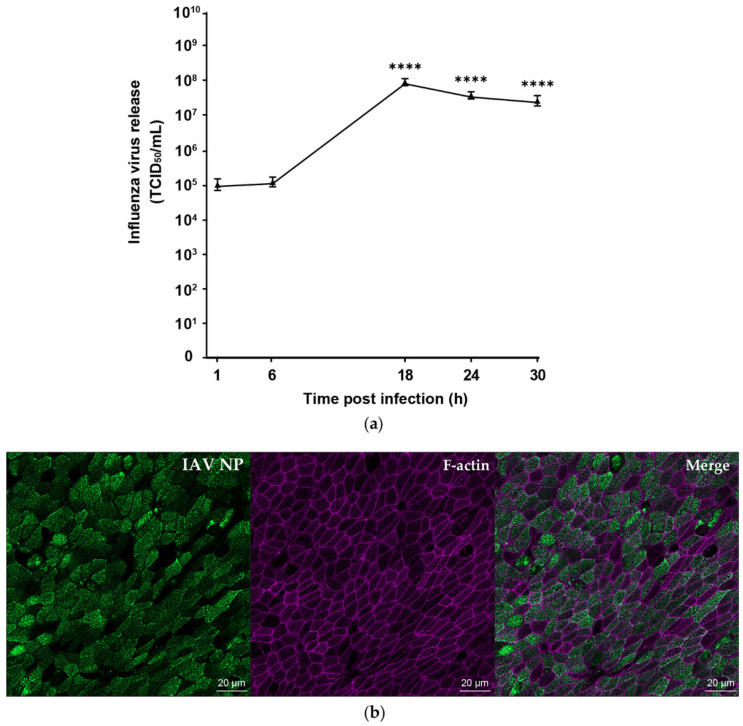
Pandemic influenza A/H1N1pdm09 infects and replicates efficiently in wdNHBE cells. (**a**). Time course of influenza virus release (TCID_50_/mL) from the apical surface of cells infected with H1N1pdm09 (MOI 1) at selected timepoints over a 30 h period postinfection. Data from three independent experiments were pooled and analyzed (Methods) using *n* = 12 biological replicates per timepoint and are presented as mean of virus release ± SEM. Statistical significance of viral titres compared to the 1 h postinfection IAV level are indicated; ****, *p* < 0.0001. (**b**) Infectivity of H1N1pdm09 in wdNHBE cells 30 h postinoculation. Cells were stained IAV nucleoprotein (IAV NP, green) and phalloidin (F-actin, cytoskeleton, pink) and the Merge also shown. Maximum intensity projections of en face Z-stacks, 13 slices (12 µm) representative of three independent experiments and a 20 µm scale bar are shown.

wdNHBE cells were infected with H1N1pdm09 at MOI of 1 and apical virus titre monitored over a 30 h time period postinfection for three independent replicate experiments (Figure 2a). H1N1pdm09 replicated efficiently in wdNHBE cells exhibiting peak viral titre of 10^8^ TCID_50_/mL at 18 h postinfection. The titre decreased by 0.5 logs at 30 h postinfection (5.8 × 10^7^; *p* < 0.0001). The virus titre at 1 h and 6 h postinfection (10^5^ TCID_50_/mL) when the virus binds and penetrates the cells is slightly higher than expected, which may be due to incomplete removal of unbound virus, despite numerous washes, and also the binding of the virus to mucus on the transwell surface. This was consistent across all three experiments. In preliminary experiments, no virus was detected in basolateral media at 72 h postinfection of wdNHBE cells with H1N1pdm09. This indicated that the virus is shed on the apical surface of polarized epithelial cells, consistent with previous studies [81,82]. Immunofluorescence assays confirmed that at 30 h postinfection, the majority of wdNHBE cells were infected with H1N1pdm09, as indicated by staining with the influenza A nucleoprotein (IAV NP, Figure 2b).

### 3.3. Pandemic Influenza Damages the Pseudostratified Airway Epithelium

**Figure 3:**
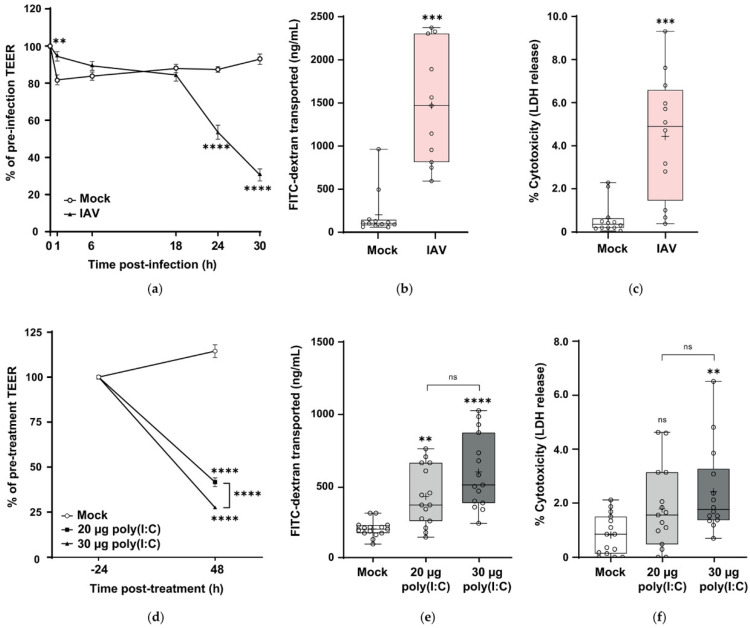
Influenza A/H1N1pdm09 and poly(I:C) damage the in vitro airway epithelium. (**a**–**c**) wdNHBE cells were inoculated with H1N1pdm09 (MO1 1) and barrier integrity compared to mock-inoculated cells. (**a**) Percentage change in transepithelial electrical resistance (TEER) readings at selected timepoints over a 30 h period postinfection for mock and H1N1pdm09-innoculated cells. (**b**) Paracellular transport of FITC-dextran (4 kDa, ng/mL) and (**c**) LDH release (percentage cytotoxicity) at 30 h postinfection for mock and H1N1pdm09-infected cells. (**d**–**f**) wdNHBE cells treated with 20 μg or 30 μg poly(I:C) were assayed 48 h post-treatment and compared to mock-treated cells for (**d**) percentage change in TEER, (**e**) FITC-dextran transported (ng/mL) and (**f**) LDH release. Data from three independent experiments for each of the H1N1pdm09 and poly(I:C) challenges were pooled and analyzed as described (Methods). For IAV TEER analyses, *n* = 36 biological replicates for each of the IAV and mock treatments per timepoint were used, apart from the 30 h mock treatment, *n* = 35. For IAV FITC-dextran and percentage cytotoxicity, *n* = 12 per treatment were analyzed. For Poly(I:C) TEERs, mock, *n* = 45; 20 μg, *n* = 30; 30 μg, *n* = 45 per group. For poly(I:C), FITC-dextran transported and cell cytotoxicity *n* = 15 per treatments were used, with the exception of the percentage cytotoxicity for 30 μg poly(I:C), *n* = 14. (**a**) and (**d**) are presented as mean ± SEM. (**b**), (**c**), (**e**) and (**f**) are presented as box and whisker plots which show the mean (+), median, interquartile range and maximum and minimum values. The ends of each box represent the upper and lower quartiles, the horizontal line within the box indicates the median value and error bars show the minimum and maximum values. Individual values are depicted by open circles. Statistical significance is indicated: ns, not significant; **, *p* < 0.01; ***, *p* < 0.001; ****, *p* < 0.0001.

To understand the effect of H1N1pdm09 on the integrity of the barrier formed by wdNHBE cells, the effect of the virus on the airway epithelium was determined by the change in TEER, paracellular permeability and LDH release (percent cytotoxicity) (Figure 3). At 6–18 h postinfection, there was no difference in percentage TEER values. During this period, the virus is undergoing virus assembly and early release of virus particles. However, at 24 h and 30 h postinfection, dramatic reductions in TEER values of 53.6 and 69.4% respectively occurred, significantly lower values than the TEERs of mock-infected cells (*p* < 0.0001; refer to Figure S3 for a graph of the individual TEER values (Ω × cm^2^) for all three independent H1N1pdm09 experiments). This significant decrease in TEER resistance was also confirmed by mixed effects modelling (*p* < 0.0001). 

From 18–30 h postinfection, higher viral replication (observed in Figure 2a) correlates with a reduction in barrier integrity of wdNHBE cells. In contrast to virus-infected cells, TEER values for mock-treated cells at 24 h and 30 h were relatively stable at 87.3 and 92.9% of pretreatment values respectively (Figure 3a).

To further characterize airway barrier integrity in response to IAV infection, the paracellular transport of FITC-dextran (4 kDa) across the epithelium was measured. Paracellular transport is regulated by AJCs, hence increased FITC-dextran transport from the apical to basolateral compartment of the transwell is an indicator of “leaky” or damaged junctions [83]. At 30 h postinfection, FITC-dextran transported across IAV-infected cells was 11.4-fold higher than for mock-treated cells (*p* < 0.001; Figure 3b), indicative of increased epithelium permeability.

Influenza A virus induces cell death [84,85]. Hence, we determined the cytotoxicity of H1N1pdm09 to wdNHBE cells by measuring the apical release of LDH 30 h postinfection. LDH is a soluble cytoplasmic enzyme that is released from the cytoplasm upon damage to the plasma membrane and is directly proportional to the number of cells undergoing apoptosis or necrosis [86,87]. At 30 h postinfection, cell death due to H1N1pdm09 was 7.1-fold greater for IAV-infected cells compared to cells treated with media alone (*p* < 0.001; Figure 3c). TUNEL staining also showed a greater number of apoptotic cells in wdNHBE cells infected with pandemic influenza compared to mock-infected cells (Figure S4). Therefore, H1N1pdm09 damage to the in vitro airway epithelium is characterized by a reduced TEER, increased paracellular flux and increased cell death.

In order to compare the immune response of wdNHBE cells to different immunostimulants, we challenged our cells with poly(I:C) at 20 μg and 30 μg and evaluated barrier integrity as for IAV-infected cells. At 48 h poststimulation, TEER values were significantly reduced in a dose-dependent manner in comparison to mock-treated cells (*p* < 0.0001; Figure 3d). Similar to the H1N1pdm09 treatment, a 72.5% reduction in TEER was recorded for 30 ug poly(I:C) and a 58.4% reduction for the 20 μg treatment. In contrast, the TEER of mock-treated cells increased by 14.5%. A graph of the individual TEER values (Ω × cm^2^) for all three, independent poly(I:C) experiments is also provided (Figure S5). The effect of poly(I:C) on paracellular flux was not as great as for H1N1pdm09. At 30 μg and 20 μg poly (I:C), FITC-dextran transported across the airway epithelium was 3-fold and 2-fold greater respectively compared to control cells (*p* < 0.0001 and *p* < 0.01 respectively; Figure 3e). Similarly, poly(I:C) was not as cytotoxic as H1N1pdm09. At the higher, 30 μg poly(I:C) dose, cell death was 3-fold higher than for control cells (*p* < 0.01; Figure 3f). However, there was no difference in cytotoxicity between the 20 μg poly(I:C) and mock treatments. In summary, both H1N1pdm09 and poly(I:C) had a detrimental effect on the integrity of the in vitro pseudostratified airway epithelium.

### 3.4. Pandemic Influenza Dissociates ZO-1 from Apical Junction Complexes

**Figure 4:**
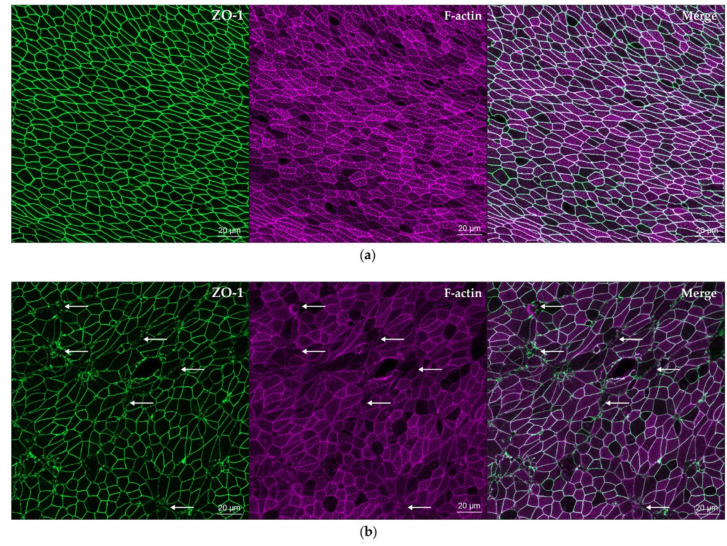
Influenza A/H1N1pdm09 damages epithelial tight junctions and the cytoskeleton of wdNHBE cells. Cells grown on transwells were (**a**) inoculated with ALI media (mock) or with (**b**) IAV H1N1pdm09 (MOI 1). At 30 h postinfection, cells were fixed in 4% PFA and stained with ZO-1 (green) and phalloidin (F-actin, pink). (**a**) While ZO-1 formed intact perijunctional belts around each cell in the mock treated cells, (**b**) ZO-1 was disrupted in IAV-infected cells (arrows, selected cells shown). Similarly, F-actin filaments were intact in mock cells (**a**), but (**b**) disrupted by IAV (arrows, selected cells shown). The colocalization of the ZO-1 perijunctional belt and its attachment to the cytoskeleton are shown by the Merge for (**a**) mock and (**b**) IAV-infected cells. Maximum intensity projections of en face Z-stacks, 19 slices (9 µm) imaged using a 40x oil immersion objective are representative of three independent experiments. A 20 µm scale bar indicates image magnification.

Influenza A viruses damage apical junctional complexes and alter the cell cytoskeleton and morphology of airway epithelial cells [34,35,83]. To investigate these characteristics in our model we performed immunofluorescence assays on mock and H1N1pdm09-infected wdNHBE cells 30 h postinfection (Figure 4). We characterized the distribution of the ZO-1 adapter protein that links tight junctions and adherens junctions to the cell cytoskeleton [88]. Phalloidin was used to stain F-actin filaments of the cytoskeleton. In mock infected wdNHBE cells, ZO-1 forms an intact apical circumferential belt (also known as the perijunctional actomyosin ring) around the plasma membrane of each cell (Figure 4a). In sharp contrast, ZO-1 was disrupted and discontinuous in many IAV H1N1pdm09-infected cells, suggestive of damage to AJCs and the epithelium (Figure 4b). These results were consistent with phalloidin staining of F-actin filaments of the cell cytoskeleton. F-actin filaments are intact in mock-infected cells (Figure 4a) but disrupted in IAV-infected cells (Figure 4b). Merged images show that the disruption of ZO-1 and F-actin of the cytoskeleton occurs in the same cells in the in vitro airway epithelium (Merge, Figure 4b). Furthermore, IAV-infected cells show evidence of cell distension. While H1N1pdm09 disrupted ZO-1 and F-actin in wdNHBE cells, this was not observed in cells treated with 30 μg poly(I:C) (Figure S6).

### 3.5. Pandemic Influenza Reduces MUC5B Expression in wdNHBE Cells

**Figure 5:**
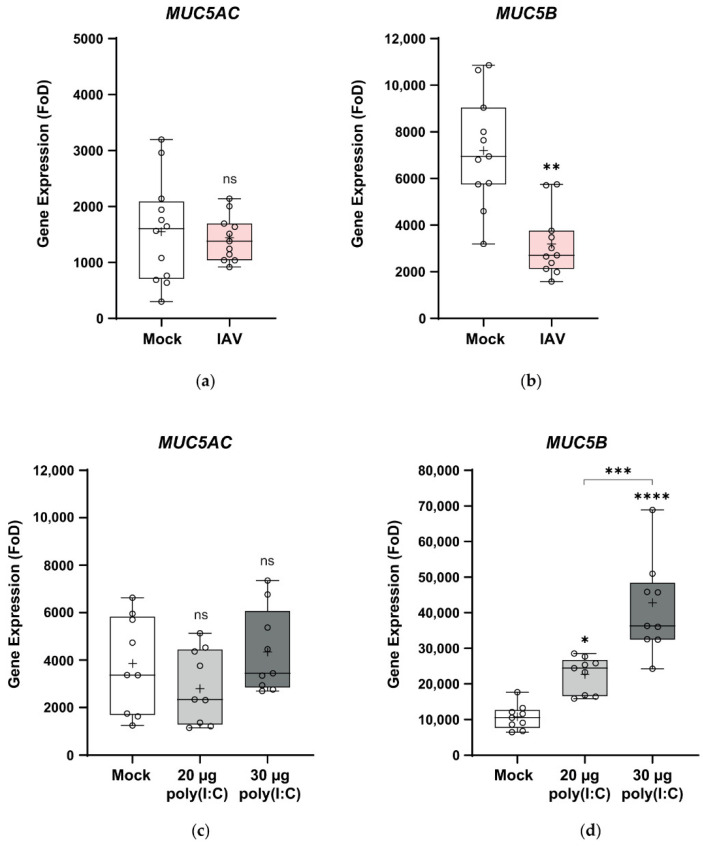
Expression of the *MUC5AC* and *MUC5B* genes in response to H1N1pdm09 and poly(I:C). The expression of *MUC5AC* and *MUC5B* in airlifted NHBE cells was assayed by RT-qPCR. (**a**) *MUC5AC* and (**b**) *MUC5B* expression 30 h postinfection with H1N1pdm09 (IAV) versus mock-inoculated cells and (**c**) *MUC5AC* and (**d**) *MUC5B* expression 48 h post-treatment with 20 μg and 30 μg poly(I:C) in comparison to mock-treated cells. Gene expression was normalized to *GAPDH* and is shown as fold over detectable (FoD), where the minimum level detectable was set at 40 cycles. Data from three independent experiments were pooled for each of IAV and poly(I:C) and are presented as box and whisker plots which show the mean (+), median, interquartile range and maximum and minimum values. The ends of each box represent the upper and lower quartiles, the horizontal line within the box indicates the median value and error bars show the minimum and maximum values. Individual values are depicted by open circles. IAV data: Mock, *n* = 12; IAV, *n* = 11 (*MUC5AC*) and Mock, *n* = 11; IAV, *n* = 11 for *MUC5B*; poly(I:C) data *n* = 9 biological replicates per treatment. Statistical significance: ns, not significant; *, *p* < 0.05; **, *p* < 0.01; ***, *p* < 0.001; ****, *p* < 0.0001.

The mucosal barrier is a major component of the innate immune system in the lungs [89,90]. MUC5AC and MUC5B are the major secreted mucins and play a critical, though poorly understood role in airway defense and mucociliary clearance [91,92]. Therefore, we analyzed the effect of H1N1pdm09 on the expression of these genes by RT-qPCR (Figure 5a). IAV had no effect on the expression of the *MUC5AC* goblet cell marker in wdNHBE cells (Figure 5a). In contrast, *MUC5B* was suppressed more than 2-fold in H1N1pdm09-inoculated cells (*p* < 0.01; Figure 5a).

Poly(I:C) treatment also had no effect on *MUC5AC* expression when compared to mock-treated cells (ns, Figure 5b), consistent with pandemic IAV. In contrast, poly(I:C) had a dose-dependent effect on *MUC5B* expression with 2-fold and 4-fold higher expression in wdNHBE cells treated with 20 μg and 30 μg poly(I:C) respectively compared to mock-treated cells (20 μg poly(I:C): *p* < 0.05; 30 μg: *p* < 0.0001; Figure 5b). 

### 3.6. Pandemic Influenza and Poly(I:C) Upregulate the Expression of Selected Cytokines, Chemokines and Antiviral Genes

We used RT-qPCR to determine whether wdNHBE cells produced an innate immune response to IAV H1N1pdm09 in vitro. Genes were selected based on their response to IAV infection with H1N1pdm09 and/or H5N1 subtypes as reported for in vitro and/or in vivo studies [58,62,93,94,95,96,97,98]. Genes assayed included: the chemokine-encoding *C-X-C motif chemokine ligand 10 (CXCL10)*, also known as *Interferon γ-induced protein 10* (*IP10*), *C-C motif chemokine ligand (CCL5*), alias *Regulated on activation, normal T cell expressed and secreted (RANTES)*, *C-C motif chemokine ligand 2 (CCL2),* also known as *Monocyte chemoattractant protein-1 (MCP1), C-C motif chemokine ligand 2 (CCL3),* alias *Macrophage inflammatory protein 1-*α *(MIP1α*), and *C-X-C motif chemokine ligand 8 (CXCL8),* also known as *Interleukin 8 (IL8)*. Proinflammatory cytokine-encoding genes characterized included: *Tumor necrosis factor (TNF), known as TNF-α*, *Interleukin 1 β (IL1B), Colony stimulating factor 2 (CSF2),* also referred to as *Granulocyte-macrophage colony stimulating factor* (*GMCSF)* and the potent proinflammatory *Interleukin 6 (IL6).* Expression of the anti-inflammatory-encoding *Interleukin 10 (IL10)* and antiviral-encoding *Interferon β 1 (IFNB1)* and *Radical S-adenosyl methionine domain containing 2 (RSAD2*), commonly known as *virus inhibitory protein, endoplasmic reticulum-associated, IFN-inducible*; *viperin* [99] was also characterized.

All genes assayed were upregulated in H1N1pdm09-infected versus mock-inoculated cells (Table 1; Figure S7). *MUC5AC* and *MUC5B* values (Figure 5) have also been included in Table 1. Notably, there was a difference in the magnitude of the response to pandemic influenza with four different levels of fold-change observed. The highest fold-increases in expression were recorded for *CXCL10* ( > 13,000-fold) and the antiviral-encoding *IFNB1* ( > 5500-fold). *CCL5* and *RSAD2* expression were also significantly upregulated in response to IAV ( > 650 and >350 fold respectively). *IL10*, *TNF*, *MIP1α* and *IL6* exhibited a 26–53-fold increase in expression compared to mock-inoculated cells. In contrast, the magnitude of response to IAV-infection was lower for the *CCL2*, *CXCL8*, *CSF2* and *IL1B* genes (2–8-fold increases). The increased expression of these cytokine, chemokine and antiviral genes suggests that innate immune responses are activated in our wdNHBE cells in response to pandemic influenza. 

**Figure 6:**
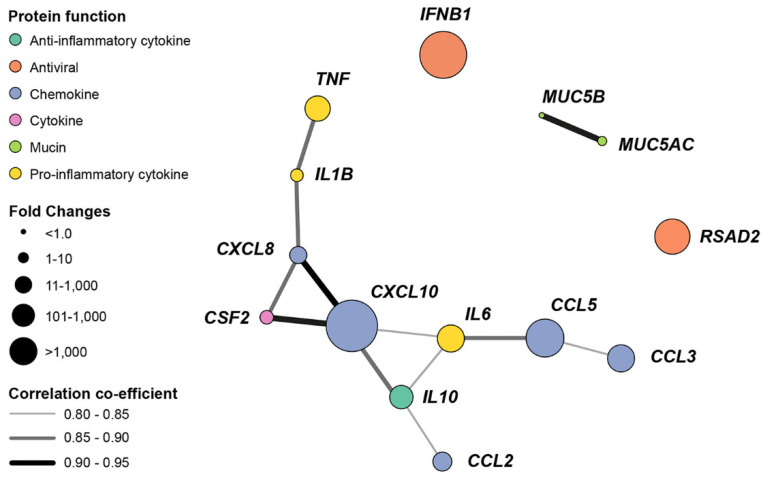
Pandemic IAV H1N1pdm09 upregulates the expression of cytokine, chemokine and antiviral genes in wdNHBE cells. The gene coexpression network depicts nodes representing the assayed genes, node size represents the magnitude of fold-change and edges (lines linking nodes) indicate the strength of the coexpression as quantified by the Pearson Correlation Coefficient. The threshold for visualizing coexpression was set at 0.80. Gene naming follows the HUGO Gene Nomenclature Committee (HGNC) [100].

This relationship was explored by the generation of a gene coexpression network (Figure 6) based on gene copy numbers (Table S4). The relationship between pro- and anti-inflammatory cytokines and chemokines genes is highlighted by the network connectivity. Similarly, association between mucin gene expression is shown. In contrast, the expression of the antiviral *IFNB1* and *RSAD2* were not linked to other genes.

The induction of innate immune response genes in wdNHBE cells was also investigated by the stimulation of wdNHBE cells with 20 µg or 30 µg poly(I:C) (Figure S8). In contrast to IAV, poly(I:C) had no effect on *CSF2* expression. While the lower, 20 μg dose of poly(I:C) was sufficient to upregulate *IL6* and *CXCL8* expression, *TNF* and *IL1B* were only induced by 30 μg of poly(I:C) compared to mock-treated cells.

### 3.7. Pandemic Influenza Stimulates the Secretion of Cytokines and Chemokines in wdNHBE Cells

**Figure 7:**
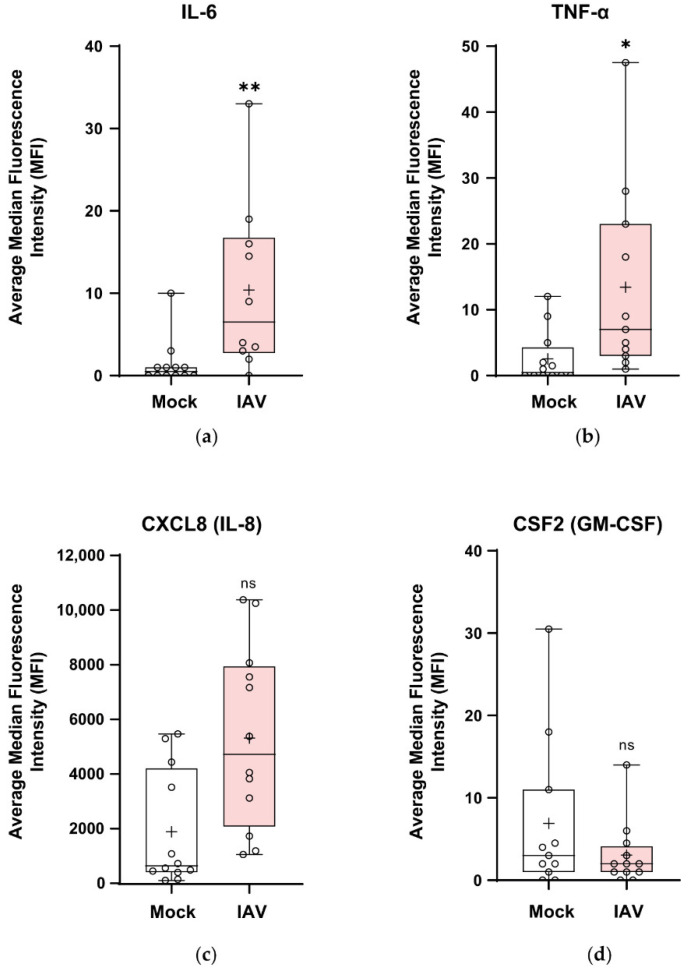
The apical secretion of cytokine and chemokines by wdNHBE cells in response to infection with IAV H1N1pdm09. At 30 h postinfection, the proinflammatory cytokines IL-6, TNF-α, CSF2 and the CXCL8 chemokine secreted into the apical compartment by wdNHBE inoculated with H1N1pdm09 or mock-infected were assayed by multiplex immunoassays. (**a**) IL-6, (**b**) TNF-α, (**c**) CXCL8 and (**d**) CSF2. ALI media was added to the upper surface and the supernatant collected after a 15 min incubation. Data was pooled from three independent experiments and the average of the median fluorescence intensity (MFI) presented as box and whisker plots showing the mean (+), median, interquartile range and maximum and minimum values. The ends of each box represent the upper and lower quartiles, the horizontal line within the box shows the median value and error bars indicate the minimum and maximum values. Individual values are depicted by open circles. IAV apical cytokines: *n* = 12 for all treatments, with the exception of IAV, IL-6 (*n* = 10); IAV TNF-α (*n* = 11) and Mock CSF2 (*n* = 11); ns, not significant; *, *p* < 0.05; **, *p* < 0.01.

To determine whether gene expression was correlated with protein secretion, multiplex immunoassays for IL-6, TNF-α, CXCL8 (IL-8) and CSF2 (GM-CSF) were performed on media harvested from the apical (Figure 7) and basolateral compartments (Figure S9) at 30 h postinfection of wdNHBE cells with H1N1pdm09. Apical secretion levels of IL-6 and TNF-α after a 15 min period were >8–12-fold higher in IAV-infected versus mock cells (Figure 7a,b), with IL-8 more than 2-fold higher (Figure 7c). In contrast, while CSF2 trended lower in IAV-infected cells, it was not significantly different to mock levels (Figure 7d). IL-1β was secreted at the minimum limit of detection (data not shown). Cytokine and chemokine secretion into the lower compartment of transwells over a 6 h period from 24 to 30 h postinfection exhibited similar trends to apical supernatants, although protein levels were generally reduced apart from IL-8 (Figure S9).

Multiplex immunoassays for IL-6, TNF-α, CXCL8, CSF2 and IL-1β were also performed for apical and basolateral media from the 20 μg and 30 μg poly(I:C) treatments (Figure S10). Trends were analogous to the response to IAV, apart from CSF2, which was stimulated, rather than suppressed by poly(I:C). The apical secretion of IL-1β, IL-6 and CSF2 was only higher for 30 μg poly(I:C) compared to mock-treated cells. In contrast, TNF-α and CXCL8 levels were greater than mock cells for both poly(I:C) doses. Cytokine and chemokine secretion in basolateral media over a 48 h period exhibited similar trends to apical media. Therefore, airlifted NHBE cells secrete cytokines and chemokines in response to both H1N1pdm09 and poly(I:C) immune system challenges. However, under experimental conditions used and properties measured, IAV elicits a much stronger inflammatory response than poly(I:C) in wdNHBE cells.

## 4. Discussion

We show that well-differentiated NHBE cells grown at the air-liquid interface are an anatomically and physiologically relevant in vitro model to investigate changes in the airway epithelium in response to H1N1pdm09 and the dsRNA viral analogue, poly(I:C). Our model recapitulated the in vivo response of the human airway epithelium to influenza in vitro. The cytopathic effect of H1N1pdm09 included damage to the airway epithelium, the induction of innate immune responses including the expression of pro- and anti-inflammatory cytokines and chemokines and antiviral genes and proteins, consistent with pulmonary host defense. To our knowledge, this is the first study that has shown ZO-1 damage in wdNHBE cells treated with H1N1pdm09. 

Additionally, the downregulation of *MUC5B*, which plays an important role in mucociliary clearance suggests another mechanism of pathogenesis induced by the virus. These results form the basis for using the ALI model for studying host–pathogen interactions and for testing therapeutics against newly emerged and re-emerging respiratory pathogens. 

The striking effect of H1N1pdm09 on ZO-1 and the disruption of the apical perijunctional actomyosin ring of the cytoskeleton highlights IAV damage to the 3D architecture of cells within the pseudostratified epithelium and is consistent with reduced TEER, increased paracellular flux across the epithelium and increased apoptosis. The pattern of ZO-1 damage in our wdNHBE cells infected with pandemic IAV is similar to that of the immortalized human bronchial epithelial cell line (16HBE14o-) infected with tissue culture adapted influenza virus A (H1N1) [83]. It also mimics ZO-1 dissociation from TJs caused by rhinovirus infection of 16HBE14o- cells, human airway epithelial cells grown at ALI and in mice in vivo [83,101]. ZO-1 dissociation is also consistent with the in vivo airway epithelium of asthmatic patients [32]. While poly(I:C) disrupted ZO-1 in cell membranes of immortalized 16HBE14o- cells [71], this was not observed in our primary wdNHBE cells. This may reflect differences between primary and immortalized cells and/or different concentrations and preparations of poly(I:C). 

Airway protection is provided by a broad-spectrum of antimicrobial factors in mucus, e.g., lysozyme, β-defensins, cathelicidin, lactoferrin, elafin, secretory leukocyte peptidase inhibitor (SLPI) and mucins [102,103,104]. However, the precise roles of mucins, in particular, MUC5AC and MUC5B, the major secretory mucins in the human airway epithelium are still to be determined. Optimal airway defense requires a balance between mucus production and clearance [92]. In our wdNHBE model, mucus removal does not occur due to the closed nature of the transwell system. However, differences in mucin gene expression for H1N1pdm09 and poly(I:C) compared to mock-inoculated cells demonstrated that wdNHBE cells respond to these immune system challenges. Differences in mucus composition and the relative abundance of MUC5AC and MUC5B can alter mucus gel viscosity, promoting pathogen growth, rather than their removal [92,105]. Hence, the significant increase in *MUC5B* expression in response to poly(I:C) but decrease in response to H1N1pdm09 and unchanged *MUC5AC* for both immune system stimulants was intriguing. Whilst the increase in *MUC5B* in response to poly(I:C) in our study was consistent with Lever and colleagues, we did not observe an increase in *MUC5AC* expression [70]. Additionally, different IAV strains (seasonal and pandemic) and different subtypes (H1N1 and H3N2) induced different levels of *MUC5AC* expression in an NCI-H292 human pulmonary mucoepidermoid carcinoma cell line [106]. Seasonal strains were stronger inducers of *MUC5AC* expression than pandemic strains and H3N2 generally had a greater effect on expression than H1N1 [106]. The differences observed in *MUC5AC* expression in our study may reflect the use of normal primary cells versus immortalized cancer lines, the use of different virus preparations and MOIs, different concentrations and/or preparations of poly(I:C) and a difference between cell donors.

Once the mucus layer of the airway epithelium has been penetrated, pathogens such as IAV attach to cell-surface receptors (α2,3- and/or α2,6- sialic acids), enter and replicate in epithelial cells. The virus egresses and spreads to other nonimmune and immune cells [56]. Although our model lacks immune cells, wdNHBE cells mount an innate immune response to both IAV and poly(I:C), consistent with separate studies of these stimulants [62,70]. The body’s natural defense mechanism of inflammation which promotes cell repair and healing [107] was mimicked in our wdNHBE model, with the increased expression of proinflammatory cytokines and chemokines such as *TNF*, *IL1B*, *IL6*, *CXCL8* and *CXCL10*. This suggests that wdNHBE cells recognize IAV and poly(I:C) through binding to pattern recognition receptors (PRRs) such as the Toll-like receptors (TLR3, TLR7, and TLR8), retinoic acid–inducible gene I (RIG-I) and melanoma differentiation–associated protein-5 (MDA-5), triggering innate immune response signaling cascades as occurs in vivo [22,54,55,108,109,110,111,112,113,114]. Antiviral, pro- and anti-inflammatory cytokines and chemokines are then upregulated in the host [27,56,115].

Key genes upregulated in our H1N1pdm09 in vitro challenge model, mimic the innate immune and inflammatory response in human patients in vivo infected with the 2009 pandemic IAV. These include *IFNB1*, *CXCL10*, *IL10*, *TNF* and *RSAD2*. The more than 5500-fold increase in *IFNB1*, which encodes the antiviral type I interferon, IFN-β, confirms the host recognition of IAV and the host-defense response to the virus in the airway epithelium in vitro, as observed by Chan et al., [58]. Interestingly, the kinetics and magnitude of IFN-β secretion and hence the early innate and antiviral responses can vary for different influenza A subtypes [116]. IFN-α/β stimulates the activation of hundreds of IFN-stimulated genes (ISGs, e.g., *CXCL10*, *RSAD2*, etc.), the first line of defence against viral infection [116,117,118,119,120]. This is observed in our model with the dramatic, 13,000-fold increase in *CXCL10* expression in response to H1N1pdm09 infection, consistent with differentiated human airway epithelial cells infected with a Hong Kong isolate of the A/H1N1 2009 pandemic strain [59], wdNHBE cells infected with pandemic IAV strains from a fatal (A/KY/180/10) and nonfatal (A/KY/136/09) case [62] and in patients infected with pandemic IAV [93,121,122,123]. Upregulation of *CXCL10* also occurs in vitro in the A549 human lung alveolar adenocarcinoma response to IAVs [124]. CXCL10 is a key chemokine responsible for early response to viral infection, is associated inflammation, e.g., asthma [125] and is a biomarker that predicts disease severity and pathogenesis [126,127,128]. The impact of *CXCL10* is reflected in the gene coexpression network, with the increased expression of a number of other chemokines, cytokines and the anti-inflammatory *IL10* gene. *CXCL10* is upregulated in response to other respiratory viruses, including RSV, rhinovirus and H5N1 [24,77,129]. However, while CXCL10 is protective in SARS-CoV infection [126], its precise role in H1N1pdm09 infection is unclear.

Increased levels of cytokines and chemokines are detected in the sera of patients with pandemic influenza [130], consistent with our model. In particular, increased levels of the proinflammatory IL-6 and the anti-inflammatory IL-10 which protects host tissue from damage during acute inflammatory responses are observed [93,95,121,131,132,133]. Elevated levels of *TNF* and *IL1B* [134] are also detected [93,122,123]. TNF-α stimulates IL-1β which exacerbates lung injury during severe influenza but may also have a vital role in lung repair after infection [130]. Other cytokines and chemokines elevated in sera of patients include the chemoattractants CXCL8 [93,131] and CCL2 [121,122,123]. Increased expression and/or secretion of these and other chemoattractants such as *CCL5* and *CCL3* were observed in our wdNHBE model, consistent with other in vitro lung cell culture studies [62,96,135]. Hence, cell-mediated immunity is triggered in wdNHBE cells, as occurs in vivo [116,119].

The more than 350-fold induction of the antiviral-encoding *RSAD2 (viperin*) gene in our pandemic IAV-infected wdNHBE cells confirms the antiviral response of the airway epithelium in vitro. It is consistent with increased *RSAD2* expression in wdNHBE cells infected with the pandemic influenza strains A/KY/180/10 and A/KY/136/09 [62] and in immortalized NCI-H441 [96] and A549 cells [124]. RSAD2 has multiple modes of antiviral activity. It catalyzes the conversion of cytidine triphosphate (CTP) to 3ʹ-deoxy-3′,4ʹ-didehydro-CTP (ddhCTP) which prematurely terminates *RNA-dependent RNA polymerase* (*RdRp*) of selected viruses but does not interfere with host RNA and DNA polymerases [136]. Hence, it reduces the replication of a range of RNA and DNA viruses including IAVs, rabies virus, HIV, West Nile virus, Zika virus, dengue virus and hepatitis C virus [136,137,138]. RSAD2 also restricts the release (budding) of IAV H1N1 and other viruses by disrupting lipid raft microdomains on the plasma membrane of host cells [99,139,140]. RSAD2 also plays a role in the activation of T-cells and T-cell-receptor-mediated activation of NF-κB and activating protein 1 (AP-1), key transcription factors in the expression of proinflammatory cytokines [140]. Hence, RSAD2 is a multifunction antiviral that has a vital role in combatting viruses such as IAV.

Airlifted primary NHBE cells grown on transwells at the air-liquid interface are the gold standard for bronchial epithelial cell culture [141]. This model provided an excellent system to investigate innate immune responses to H1N1pdm09 and poly(I:C) in the airway epithelium in vitro. Future studies could investigate the addition of other cell types including fibroblasts, endothelial smooth muscle cells and immune cells, e.g., neutrophils, to better reproduce the human airway in vitro and will undoubtably provide further information on host–pathogen interactions in the lung.

## 5. Conclusions

Airlifted NHBE cells recapitulated the pseudostratified airway epithelium in vivo, with the formation of AJCs, mucus secretion and the coordinated beating of ciliated cells. The disruption of the airway epithelium by IAV H1N1pdm09 and poly(I:C), plus the induction of the innate immune response and antiviral, and pro- and anti-inflammatory genes demonstrated the viability of this model to investigate pandemic influenza. The disassembly of the AJC by H1N1pdm09 as shown by damage to ZO-1 suggests that the virus can penetrate the epithelium and hence produce systemic infection. The use of multiple immune system challenges enabled the identification of differential responses of wdNHBE cells to H1N1pdm09 and poly(I:C). The reduction of *MUC5B* in response to IAV is indicative of impaired mucociliary clearance of IAV which may contribute to the severity of pandemic influenza in vivo. Future studies will focus of the use of this model to investigate zoonotic, emerging infectious viruses with pandemic potential including the SARS-CoV-2 coronavirus currently sweeping the world today.

## Figures and Tables

**Table 1:** Fold change (FoD) in gene expression 30 h postinfection with IAV H1N1pdm09- versus mock-inoculated wdNHBE cells.

Gene	Mock FoD ±SEM	IAV FoD ±SEM	Fold Change (IAV FoD/Mock FoD), Significance, *p*-value	Protein Function
*CXCL10 (IP10)*	151.5 ± 37.5	2,007,204 ± 212,503	13,249; ****, *p* < 0.0001	Antimicrobial, proinflammatory chemokine
*IFNB1*	10.5 ± 1.8	57,944 ± 8,666	5,513; ****, *p* < 0.0001	Antiviral cytokine
*CCL5 (RANTES)*	462.9 ± 84.6	305,095 ± 17,110	659; ****, *p* < 0.0001	Chemokine
*RSAD2 (viperin)*	558.8 ± 42.5	209,055 ± 14,422	374; ****, *p* < 0.0001	Antiviral
*IL6*	46.3 ± 9.6	2,453 ± 175	53; ****, *p* < 0.0001	Proinflammatory cytokine
*CCL3 (MIP1α)*	2.2 ± 0.5	113.4 ± 10.4	51; ****, *p* < 0.0001	Chemokine
*TNF*	83.3 ± 19.5	3,177 ± 315	38; ****, *p* < 0.0001	Proinflammatory cytokine
*IL10*	5.7 ± 0.8	148.6 ± 19.4	26; ****, *p* < 0.0001	Anti-inflammatory cytokine
*CCL2 (MCP1)*	211.8 ± 39.8	1,814 ± 284	8.6; ****, *p* < 0.0001	Chemokine
*CXCL8 (IL8)*	90,609 ± 26,475	510,793 ± 64,995	5.6; ****, *p* < 0.0001	Chemokine
*CSF2 (GMCSF)*	143.2 ± 28.6	347.9 ± 46.0	2.4; **, *p* = 0.001	Cytokine
*IL1B*	2,645 ± 471	5,679 ± 890	2.1; **, *p* = 0.0064	Potent Proinflammatory cytokine
*MUC5AC*	1,556 ± 264	1,431 ± 122	0.9; ns, *p* = 0.6815	Mucin
*MUC5B*	7,207 ± 718	3,195 ± 423	0.4; ***, *p* = 0.0001	Mucin

## Supplementary Materials

The following are available online at https://www.mdpi.com/1999-4915/12/6/679/s1, Table S1: TaqMan Probes used for RT-qPCR analyses, Video S1: Beating of cilia on the surface of ciliated cells of the wdNHBE epithelium, Figure S1: Transepithelial electrical resistance (TEER) readings of wdNHBE cells, Table S2: TEER readings (Ω & Ω × cm2) wdNHBE cells - IAV H1N1pdm09, Table S3: TEER readings (Ω & Ω × cm2) wdNHBE cells - poly(IC), Figure S2: FACS analysis of α-2-3 and α-2-6-linked sialic acids on the surface of wdNHBE cells, Figure S3: Transepithelial electrical resistance (TEER) readings for three independent experiments of Mock vs IAV H1N1pdm09 inoculated wdNHBE cells, Figure S4: Apoptosis in wdNHBE cells infected with pandemic IAV H1N1pdm09, Figure S5: Transepithelial electrical resistance (TEER) readings for three independent experiments of wdNHBE cells treated with 20 µg and 30 µg poly(I:C) vs mock-treated cells, Figure S6: Immunofluorescence assays of poly(I:C)-treated wdNHBE cells, Figure S7: Expression of cytokines, chemokines and antiviral genes in wdNHBE cells in response to pandemic influenza infection, Table S4: Gene copy numbers for IAV H1N1pdm09 vs. mock-infected wdNHBE cells, Figure S8: Poly(I:C) stimulation of innate immune response genes in wdNHBE cells, Figure S9: The basolateral secretion of cytokines and chemokines by wdNHBE cells in response to infection with IAV H1N1pdm09, Figure S10: The apical and basolateral secretion of cytokines and chemokines by wdNHBE cells in response to poly(I:C) treatment.
