# Supplementary material for: Host–Pathogen Responses to Pandemic Influenza H1N1pdm09 in a Human Respiratory Airway Model
Source: Viruses. 2020 Jun 24;12(6):679. doi: 10.3390/v12060679 (PMC7354428; doi:10.3390/v12060679)
Supplement: Supplementary file 1 [file viruses-12-00679-s001.zip › Supplementary Files/Supplementary Files1.pdf]

Table S1: TaqMan Probes used for RT-qPCR analyses

HGNC Gene Symbol[1]	HGNC Gene Name[1]	HGNC ID[1]	Common Alias Symbol(s)[1]	Alias Name[1]	Gene Group[1]	TaqMan Probe ID	Amplicon length (bp)
CSF2	*Colony stimulating factor 2*	2434	*GM-CSF*	*Granulocyte-macrophage colony stimulating factor*	-	Hs00929873_m1	85
CCL2	*C-C motif chemokine ligand 2*	10618	*MCP-1*	*Monocyte chemoattractant protein-1*	Chemokine ligands	Hs00234140_m1	101
CCL3	*C-C motif chemokine ligand 3*	10627	*MIP1-α*	*Macrophage inflammatory protein 1-alpha*	Chemokine ligands	Hs00234142_m1	53
CCL5	*C-C motif chemokine ligand 5*	10632	*RANTES*	*Regulated on activation, normal T cell expressed and secreted*	Chemokine ligands	Hs00982282_m1	70
CXCL8	*C-X-C motif chemokine ligand 8*	6025	*IL-8*	*Interleukin 8*	Chemokine ligands, Interleukins	Hs00174103_m1	101
CXCL10	*C-X-C motif chemokine ligand 10*	10637	*IP-10*	*Interferon gamma-induced protein 10*	Chemokine ligands	Hs00171042_m1	98
GAPDH	*Glyceraldehyde-3-phosphate dehydrogenase*	4141	*GAPD*	-	-	Hs99999905_m1	122
IFNB1	*Interferon β 1*	5434	*IFF, IFB*	*Interferon, fibroblast*	Interferons	Hs01077958_s1	73
IL1B	*Interleukin 1 β*	5992	*IL1F2*	*Interleukin 1 family 2*	Interleukins	Hs01555410_m1	91
IL6	*Interleukin 6*	6018	*BSF2, HGF*	*B cell stimulatory factor 2*	Interleukins, Interferons, Interleukin 6 type family	Hs00985639_m1	66
IL10	*Interleukin 10*	5962	*CSIF*	*Cytokine synthesis inhibitory factor*	Interleukins	Hs00961622_m1	74
MUC5AC	*Mucin 5AC, oligomeric mucus / gel-forming*	7515	*MUC5*	*mucin 5*	Mucins	Hs01365616_m1	55
MUC5B	*Mucin 5B, oligomeric mucus / gel-forming*	7516	*MG1*	*Mucin glycoprotein 1*	Mucins; MicroRNA protein coding host genes	Hs00861588_m1	60
RSAD2	*Radical S-adenosyl methionine domain containing 2*	30908	*viperin*	*Virus inhibitory protein, endoplasmic reticulum associated, interferon inducible*	-	Hs00369813_m1	76
TNF	*Tumor necrosis factor*	11892	*TNFA*	*Tumor necrosis factor alpha*	Tumor necrosis factor superfamily	Hs00174128_m1	80

1. Braschi, B.;Denny, P.;Gray, K.;Jones, T.;Seal, R.;Tweedie, S.;Yates, B.;Bruford, E., Genenames.org: the HGNC and VGNC resources in 2019. *Nucleic Acids Res*, **2019**. *47*(D1): p. D786-D792. DOI: 10.1093/nar/gky930.

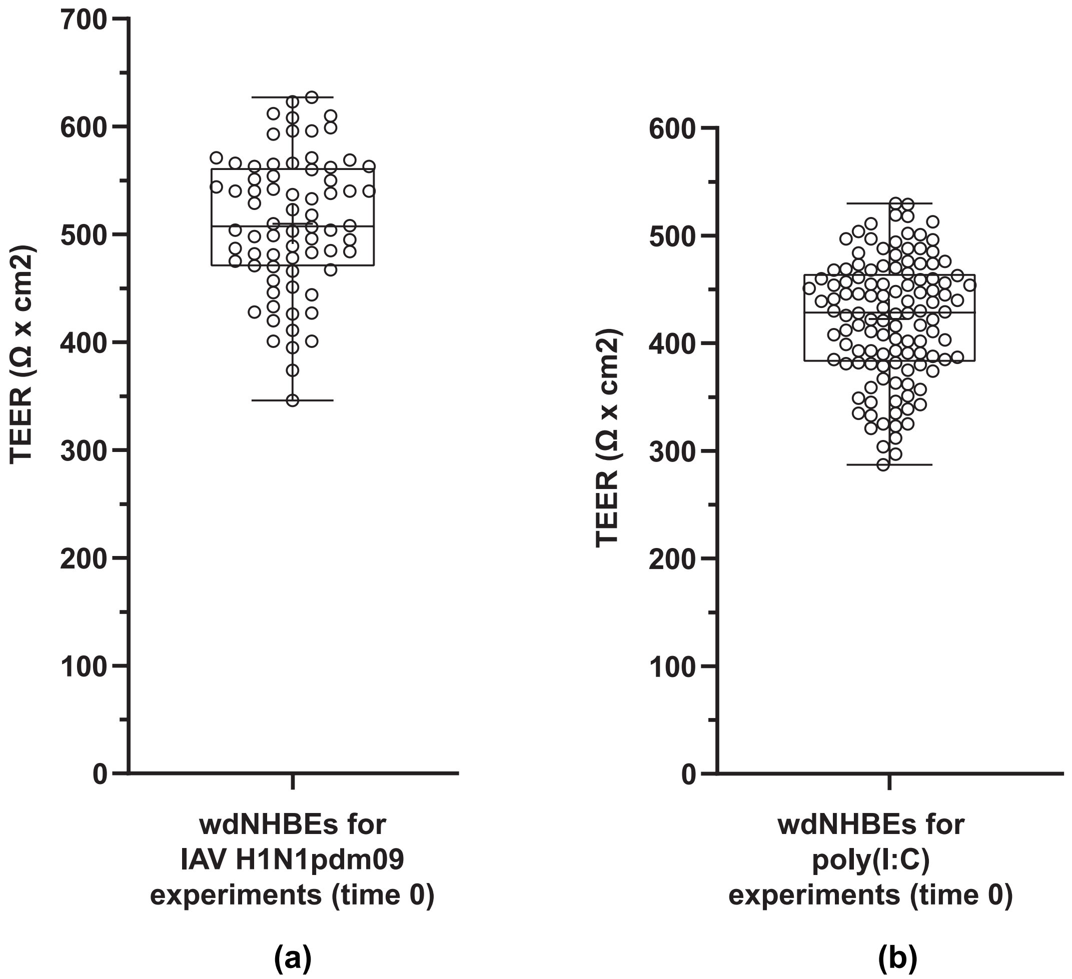

Figure S1. Transepithelial electrical resistance (TEER) readings of wdNHBE cells
Prior to infection with (**a**) IAV H1N1pdm09 or treatment with (**b**) poly(I:C), time=0 TEER values were recorded with an EVOM2. Data was pooled from three experiments for IAV H1N1pdm09 (n = 72 transwells) and three poly(I:C) experiments (n=120 transwells) was pooled (open circles) and is presented as box and whisker plots showing the mean (+), median, interquartile range and maximum and minimum values. The ends of each box represent the upper and lower quartiles, the horizontal line within the box indicates the median and error bars depict maximum and minimum TEER values.

Table S2: TEER readings (Ω & Ω x cm2) wdNHBE cells - IAV H1N1pdm09

TEER readings (Ω)

MOCK			TEER readings (Ω)					
			Pre-infection	Hours post-infection				
Treatment	Expt	Well	-1	1	6	18	24	30
Mock	a	1	1677	769	1242	1402	1308	*
Mock	a	2	1385	597	914	1061	1075	1345
Mock	a	3	1470	1025	1168	1096	1083	1301
Mock	a	4	1290	1281	1343	1357	1162	1566
Mock	a	5	1416	1161	1163	1339	1311	1340
Mock	a	6	1499	1653	1436	1455	1458	1449
Mock	a	7	1615	1671	1403	1482	1520	1488
Mock	a	8	1513	1353	1230	1503	1535	1564
Mock	a	9	1806	1465	1608	1712	1680	1072
Mock	a	10	1729	1594	1639	1560	1545	1345
Mock	a	11	1626	1069	1500	1441	1464	1267
Mock	a	12	1642	1314	1517	1529	1586	1398
Mock	b	1	1350	1145	1305	1352	1330	1394
Mock	b	2	1423	774	1094	1443	1108	1339
Mock	b	3	1245	1051	1125	1290	1092	1312
Mock	b	4	1474	1287	1264	1209	1110	1353
Mock	b	5	1716	1507	1245	1308	1308	1419
Mock	b	6	1529	1271	1177	1419	1421	1668
Mock	b	7	1636	1249	1056	1316	1372	1690
Mock	b	8	1637	1416	1144	959	1304	1117
Mock	b	9	1666	1125	1190	1335	1363	1194
Mock	b	10	1697	1203	1212	1363	1388	1466
Mock	b	11	1049	1041	1106	1221	1141	1315
Mock	b	12	1849	1692	1555	1399	1432	1163
Mock	c	1	1427	1524	1487	1369	1280	1615
Mock	c	2	1458	1406	1570	1463	1295	1630
Mock	c	3	1367	1413	1526	1555	1345	1659
Mock	c	4	1439	1266	1537	1559	1458	1762
Mock	c	5	1466	1019	1101	1255	1393	1453
Mock	c	6	1806	1608	1312	1428	1601	1609
Mock	c	7	1843	1419	1301	1536	1621	1659
Mock	c	8	1888	1863	1439	1564	1641	1642
Mock	c	9	1706	1040	1164	1163	1273	1350
Mock	c	10	1525	1089	1212	1159	1330	1273
Mock	c	11	1853	1434	1297	1222	1421	1476
Mock	c	12	1670	1163	1189	1102	1225	1297
		**AVERAGE**	**1566**	**1277**	**1299**	**1359**	**1361**	**1429**

IAV H1N1pdm09			TEER readings (Ω)					
			Pre-infection	Hours post-infection				
Treatment	Expt	Well	-1	1	6	18	24	30
H1N1pdm09	a	1	1294	1295	1602	1634	1004	451
H1N1pdm09	a	2	1273	1580	1601	1614	1471	659
H1N1pdm09	a	3	1214	1064	1251	1623	1246	647
H1N1pdm09	a	4	1604	1521	1621	1595	1153	505
H1N1pdm09	a	5	1716	1451	1241	1249	453	242
H1N1pdm09	a	6	1529	1855	1421	1302	545	281
H1N1pdm09	a	7	1636	1669	1422	715	380	220
H1N1pdm09	a	8	1637	1793	1842	1330	450	263
H1N1pdm09	a	9	1814	1375	1398	1180	523	195
H1N1pdm09	a	10	1298	1043	1505	931	351	162
H1N1pdm09	a	11	1731	1171	1479	1105	528	241
H1N1pdm09	a	12	1411	1363	1330	731	365	200
H1N1pdm09	b	1	1344	1535	1371	1345	934	782
H1N1pdm09	b	2	1132	951	1040	1153	838	854
H1N1pdm09	b	3	1461	1338	1298	1271	1078	839
H1N1pdm09	b	4	1216	1181	1289	1398	1065	746
H1N1pdm09	b	5	1569	1442	1276	1344	680	328
H1N1pdm09	b	6	1632	1379	1371	1491	872	435
H1N1pdm09	b	7	1464	1536	1271	1364	858	406
H1N1pdm09	b	8	1448	1526	1195	1232	744	355
H1N1pdm09	b	9	1713	1022	1223	1353	889	380
H1N1pdm09	b	10	1311	1275	1105	834	462	205
H1N1pdm09	b	11	1707	1558	1395	840	606	211
H1N1pdm09	b	12	1702	1471	1334	1305	698	197
H1N1pdm09	c	1	1536	1511	1580	1436	1291	649
H1N1pdm09	c	2	1510	1339	1387	1243	1098	919
H1N1pdm09	c	3	1545	899	1184	1040	895	719
H1N1pdm09	c	4	1198	1021	1145	1001	856	969
H1N1pdm09	c	5	1539	1650	1266	1378	924	491
H1N1pdm09	c	6	1503	1392	1179	1313	747	323
H1N1pdm09	c	7	1483	1496	1229	1222	527	285
H1N1pdm09	c	8	1899	1249	1339	1683	1131	388
H1N1pdm09	c	9	1723	1508	1233	1249	864	335
H1N1pdm09	c	10	1584	1110	1189	1096	618	262
H1N1pdm09	c	11	1796	1753	1476	1340	692	265
H1N1pdm09	c	12	1648	1629	1367	1153	676	287
		**AVERAGE**	**1523**	**1388**	**1346**	**1253**	**792**	**436**

TEER readings (Ω x cm2)

Transwell area 0.33 cm^2

			TEER readings (Ω x cm^2)					
			Pre-infection	Hours post-infection				
Treatment	Expt	Well	-1	1	6	18	24	30
Mock	a	1	554	254	410	463	432	
Mock	a	2	457	197	302	350	355	444
Mock	a	3	485	338	386	362	358	429
Mock	a	4	426	423	443	448	384	517
Mock	a	5	467	383	384	442	433	442
Mock	a	6	495	546	474	480	481	478
Mock	a	7	533	552	463	489	501	491
Mock	a	8	499	447	406	496	506	516
Mock	a	9	596	484	531	565	554	354
Mock	a	10	571	526	541	515	510	444
Mock	a	11	537	353	495	476	483	418
Mock	a	12	542	434	501	505	523	461
Mock	b	1	446	378	431	446	439	460
Mock	b	2	470	255	361	476	366	442
Mock	b	3	411	347	371	426	360	433
Mock	b	4	487	425	417	399	366	447
Mock	b	5	566	497	411	432	432	468
Mock	b	6	504	420	388	468	469	551
Mock	b	7	540	412	348	434	453	558
Mock	b	8	540	467	377	316	430	369
Mock	b	9	550	371	393	441	450	394
Mock	b	10	560	397	400	450	458	484
Mock	b	11	346	344	365	403	377	434
Mock	b	12	610	558	513	462	473	384
Mock	c	1	471	503	491	452	423	533
Mock	c	2	481	464	518	483	427	538
Mock	c	3	451	466	504	513	444	548
Mock	c	4	475	418	507	515	481	582
Mock	c	5	484	336	363	414	460	480
Mock	c	6	596	531	433	471	528	531
Mock	c	7	608	468	429	507	535	548
Mock	c	8	623	615	475	516	541	542
Mock	c	9	563	343	384	384	420	446
Mock	c	10	503	359	400	383	439	420
Mock	c	11	612	473	428	403	469	487
Mock	c	12	551	384	392	364	404	428
		**AVERAGE**	**517**	**421**	**429**	**449**	**449**	**471**

			TEER readings (Ω x cm^2)					
			Pre-infection	Hours post-infection				
Treatment	Expt	Well	-1	1	6	18	24	30
H1N1pdm09	a	1	427	427	529	539	331	149
H1N1pdm09	a	2	420	521	528	533	486	218
H1N1pdm09	a	3	401	351	413	536	411	214
H1N1pdm09	a	4	529	502	535	526	381	167
H1N1pdm09	a	5	566	479	409	412	149	80
H1N1pdm09	a	6	504	612	469	430	180	93
H1N1pdm09	a	7	540	551	469	236	125	73
H1N1pdm09	a	8	540	592	608	439	148	87
H1N1pdm09	a	9	599	454	461	390	173	64
H1N1pdm09	a	10	428	344	497	307	116	54
H1N1pdm09	a	11	571	387	488	365	174	80
H1N1pdm09	a	12	466	450	439	241	120	66
H1N1pdm09	b	1	444	506	453	444	308	258
H1N1pdm09	b	2	374	314	343	381	277	282
H1N1pdm09	b	3	482	441	428	420	356	277
H1N1pdm09	b	4	401	390	425	461	352	246
H1N1pdm09	b	5	518	476	421	443	224	108
H1N1pdm09	b	6	538	455	452	492	288	144
H1N1pdm09	b	7	483	507	419	450	283	134
H1N1pdm09	b	8	478	504	394	406	245	117
H1N1pdm09	b	9	565	337	404	447	293	126
H1N1pdm09	b	10	433	421	365	275	152	68
H1N1pdm09	b	11	563	514	460	277	200	70
H1N1pdm09	b	12	562	486	440	431	230	65
H1N1pdm09	c	1	507	499	522	474	426	214
H1N1pdm09	c	2	498	442	458	410	362	303
H1N1pdm09	c	3	510	297	391	343	295	237
H1N1pdm09	c	4	395	337	378	330	282	320
H1N1pdm09	c	5	508	545	418	455	305	162
H1N1pdm09	c	6	496	459	389	433	246	107
H1N1pdm09	c	7	489	494	405	403	174	94
H1N1pdm09	c	8	627	412	442	555	373	128
H1N1pdm09	c	9	569	498	407	412	285	111
H1N1pdm09	c	10	523	366	392	362	204	87
H1N1pdm09	c	11	593	579	487	442	228	88
H1N1pdm09	c	12	544	538	451	381	223	95
		**AVERAGE**	**503**	**458**	**444**	**413**	**261**	**144**

Table S3: TEER readings (Ω & Ω x cm2) wdNHBE cells - poly(I:C)

TEER readings (Ω)

MOCK

Treatment	Expt	Well	TEER readings (Ω) Pre-treatment -24	Hours post-treatment 48
Mock	2a	1	1360	1597
Mock	2a	2	1378	1579
Mock	2a	3	1467	1534
Mock	2a	4	1311	1565
Mock	2a	5	1436	1614
Mock	2a	6	1394	1454
Mock	2a	7	1380	1695
Mock	2a	8	1470	1327
Mock	2a	9	1337	1504
Mock	2a	10	1296	1298
Mock	2a	11	1506	1564
Mock	2a	12	1606	1490
Mock	2a	13	1496	1438
Mock	2a	14	1327	1324
Mock	2a	15	1418	1487
Mock	2b	1	1026	1715
Mock	2b	2	1083	1737
Mock	2b	3	900	1543
Mock	2b	4	1099	1600
Mock	2b	5	869	1766
Mock	2b	6	1221	979
Mock	2b	7	1218	1409
Mock	2b	8	985	1634
Mock	2b	9	1174	1423
Mock	2b	10	986	1353
Mock	2b	11	1181	922
Mock	2b	12	1190	1475
Mock	2b	13	1264	1234
Mock	2b	14	1177	980
Mock	2b	15	1137	1395
Mock	2c	1	1357	1684
Mock	2c	2	1462	1736
Mock	2c	3	1156	1348
Mock	2c	4	1039	1135
Mock	2c	5	1049	1195
Mock	2c	6	1111	799
Mock	2c	7	1292	1227
Mock	2c	8	1236	1215
Mock	2c	9	1217	1104
Mock	2c	10	1503	785
Mock	2c	11	1262	1340
Mock	2c	12	1280	1363
Mock	2c	13	1375	1191
Mock	2c	14	1330	1530
Mock	2c	15	1376	1279
		**AVERAGE**	**1261**	**1390**

20 µg Poly(I:C)

Treatment	Expt	Well	TEER readings (Ω) Pre-treatment -24	Hours post-treatment 48
20 µg Poly(I:C)	2a	1	1330	539
20 µg Poly(I:C)	2a	2	1441	675
20 µg Poly(I:C)	2a	3	1418	628
20 µg Poly(I:C)	2a	4	1394	366
20 µg Poly(I:C)	2a	5	1381	799
20 µg Poly(I:C)	2a	6	1350	341
20 µg Poly(I:C)	2a	7	1573	522
20 µg Poly(I:C)	2a	8	1355	404
20 µg Poly(I:C)	2a	9	1442	401
20 µg Poly(I:C)	2a	10	1349	646
20 µg Poly(I:C)	2b	1	1345	745
20 µg Poly(I:C)	2b	2	1015	316
20 µg Poly(I:C)	2b	3	921	518
20 µg Poly(I:C)	2b	4	1045	497
20 µg Poly(I:C)	2b	5	979	458
20 µg Poly(I:C)	2b	6	973	239
20 µg Poly(I:C)	2b	7	1014	184
20 µg Poly(I:C)	2b	8	1302	273
20 µg Poly(I:C)	2b	9	1088	577
20 µg Poly(I:C)	2b	10	1065	536
20 µg Poly(I:C)	2c	1	1158	378
20 µg Poly(I:C)	2c	2	1224	679
20 µg Poly(I:C)	2c	3	1057	833
20 µg Poly(I:C)	2c	4	1158	518
20 µg Poly(I:C)	2c	5	1167	395
20 µg Poly(I:C)	2c	6	1280	395
20 µg Poly(I:C)	2c	7	1404	270
20 µg Poly(I:C)	2c	8	1167	471
20 µg Poly(I:C)	2c	9	1519	1096
20 µg Poly(I:C)	2c	10	1299	394
		**AVERAGE**	**1240**	**503**

30 µg Poly(I:C)

Treatment	Expt	Well	TEER readings (Ω) Pre-treatment -24	Hours post-treatment 48
30 µg Poly(I:C)	2a	1	1420	354
30 µg Poly(I:C)	2a	2	1410	281
30 µg Poly(I:C)	2a	3	1277	433
30 µg Poly(I:C)	2a	4	1436	247
30 µg Poly(I:C)	2a	5	1345	400
30 µg Poly(I:C)	2a	6	1569	292
30 µg Poly(I:C)	2a	7	1547	299
30 µg Poly(I:C)	2a	8	1386	400
30 µg Poly(I:C)	2a	9	1479	306
30 µg Poly(I:C)	2a	10	1480	303
30 µg Poly(I:C)	2a	11	1430	397
30 µg Poly(I:C)	2a	12	1366	403
30 µg Poly(I:C)	2a	13	1478	320
30 µg Poly(I:C)	2a	14	1528	340
30 µg Poly(I:C)	2a	15	1507	329
30 µg Poly(I:C)	2b	1	1423	221
30 µg Poly(I:C)	2b	2	1398	444
30 µg Poly(I:C)	2b	3	1133	296
30 µg Poly(I:C)	2b	4	1265	433
30 µg Poly(I:C)	2b	5	1008	319
30 µg Poly(I:C)	2b	6	1190	287
30 µg Poly(I:C)	2b	7	1153	279
30 µg Poly(I:C)	2b	8	946	206
30 µg Poly(I:C)	2b	9	1246	275
30 µg Poly(I:C)	2b	10	1098	266
30 µg Poly(I:C)	2b	11	1303	380
30 µg Poly(I:C)	2b	12	1248	266
30 µg Poly(I:C)	2b	13	1296	445
30 µg Poly(I:C)	2b	14	1186	249
30 µg Poly(I:C)	2b	15	1209	246
30 µg Poly(I:C)	2c	1	1333	468
30 µg Poly(I:C)	2c	2	1555	313
30 µg Poly(I:C)	2c	3	1432	269
30 µg Poly(I:C)	2c	4	1148	368
30 µg Poly(I:C)	2c	5	1191	906
30 µg Poly(I:C)	2c	6	1237	293
30 µg Poly(I:C)	2c	7	1245	333
30 µg Poly(I:C)	2c	8	1350	233
30 µg Poly(I:C)	2c	9	1522	272
30 µg Poly(I:C)	2c	10	1156	389
30 µg Poly(I:C)	2c	11	1376	165
30 µg Poly(I:C)	2c	12	1390	287
30 µg Poly(I:C)	2c	13	1184	342
30 µg Poly(I:C)	2c	14	1602	391
30 µg Poly(I:C)	2c	15	1294	125
		**AVERAGE**	**1328**	**330**

TEER readings (Ω x cm2)

MOCK

Transwell area	0.33	cm^2	TEER readings ($\Omega \times cm^2$) Pre-treatment	Hours post-treatment
**Treatment**	**Expt**	**Well**	**-24**	**48**
Mock	2a	1	449	527
Mock	2a	2	455	521
Mock	2a	3	484	506
Mock	2a	4	433	516
Mock	2a	5	474	533
Mock	2a	6	460	480
Mock	2a	7	455	559
Mock	2a	8	485	438
Mock	2a	9	441	496
Mock	2a	10	428	428
Mock	2a	11	497	516
Mock	2a	12	530	492
Mock	2a	13	494	475
Mock	2a	14	438	437
Mock	2a	15	468	491
Mock	2b	1	339	566
Mock	2b	2	357	573
Mock	2b	3	297	509
Mock	2b	4	363	528
Mock	2b	5	287	583
Mock	2b	6	403	323
Mock	2b	7	402	465
Mock	2b	8	325	539
Mock	2b	9	387	470
Mock	2b	10	325	446
Mock	2b	11	390	304
Mock	2b	12	393	487
Mock	2b	13	417	407
Mock	2b	14	388	323
Mock	2b	15	375	460
Mock	2c	1	448	556
Mock	2c	2	482	573
Mock	2c	3	381	445
Mock	2c	4	343	375
Mock	2c	5	346	394
Mock	2c	6	367	264
Mock	2c	7	426	405
Mock	2c	8	408	401
Mock	2c	9	402	364
Mock	2c	10	496	259
Mock	2c	11	416	442
Mock	2c	12	422	450
Mock	2c	13	454	393
Mock	2c	14	439	505
Mock	2c	15	454	422
		**AVERAGE**	**416**	**459**

20 µg Poly(I:C)

Transwell area	0.33	cm^2	TEER readings ($\Omega \times cm^2$) Pre-treatment	Hours post-treatment
**Treatment**	**Expt**	**Well**	**-24**	**48**
20 µg Poly(I:C)	2a	1	439	178
20 µg Poly(I:C)	2a	2	476	223
20 µg Poly(I:C)	2a	3	468	207
20 µg Poly(I:C)	2a	4	460	121
20 µg Poly(I:C)	2a	5	456	264
20 µg Poly(I:C)	2a	6	446	113
20 µg Poly(I:C)	2a	7	519	172
20 µg Poly(I:C)	2a	8	447	133
20 µg Poly(I:C)	2a	9	476	132
20 µg Poly(I:C)	2a	10	445	213
20 µg Poly(I:C)	2b	1	444	246
20 µg Poly(I:C)	2b	2	335	104
20 µg Poly(I:C)	2b	3	304	171
20 µg Poly(I:C)	2b	4	345	164
20 µg Poly(I:C)	2b	5	323	151
20 µg Poly(I:C)	2b	6	321	79
20 µg Poly(I:C)	2b	7	335	61
20 µg Poly(I:C)	2b	8	430	90
20 µg Poly(I:C)	2b	9	359	190
20 µg Poly(I:C)	2b	10	351	177
20 µg Poly(I:C)	2c	1	382	125
20 µg Poly(I:C)	2c	2	404	224
20 µg Poly(I:C)	2c	3	349	275
20 µg Poly(I:C)	2c	4	382	171
20 µg Poly(I:C)	2c	5	385	130
20 µg Poly(I:C)	2c	6	422	130
20 µg Poly(I:C)	2c	7	463	89
20 µg Poly(I:C)	2c	8	385	155
20 µg Poly(I:C)	2c	9	501	362
20 µg Poly(I:C)	2c	10	429	130
		**AVERAGE**	**409**	**166**

30 µg Poly(I:C)

Transwell area	0.33	cm^2	TEER readings ($\Omega \times cm^2$) Pre-treatment	Hours post-treatment
**Treatment**	**Expt**	**Well**	**-24**	**48**
Mock	2a	1	469	117
Mock	2a	2	465	93
Mock	2a	3	421	143
Mock	2a	4	474	82
Mock	2a	5	444	132
Mock	2a	6	518	96
Mock	2a	7	511	99
Mock	2a	8	457	132
Mock	2a	9	488	101
Mock	2a	10	488	100
Mock	2a	11	472	131
Mock	2a	12	451	133
Mock	2a	13	488	106
Mock	2a	14	504	112
Mock	2a	15	497	109
Mock	2b	1	470	73
Mock	2b	2	461	147
Mock	2b	3	374	98
Mock	2b	4	417	143
Mock	2b	5	333	105
Mock	2b	6	393	95
Mock	2b	7	380	92
Mock	2b	8	312	68
Mock	2b	9	411	91
Mock	2b	10	362	88
Mock	2b	11	430	125
Mock	2b	12	412	88
Mock	2b	13	428	147
Mock	2b	14	391	82
Mock	2b	15	399	81
Mock	2c	1	440	154
Mock	2c	2	513	103
Mock	2c	3	473	89
Mock	2c	4	379	121
Mock	2c	5	393	299
Mock	2c	6	408	97
Mock	2c	7	411	110
Mock	2c	8	446	77
Mock	2c	9	502	90
Mock	2c	10	381	128
Mock	2c	11	454	54
Mock	2c	12	459	95
Mock	2c	13	391	113
Mock	2c	14	529	129
Mock	2c	15	427	41
		**AVERAGE**	**438**	**109**

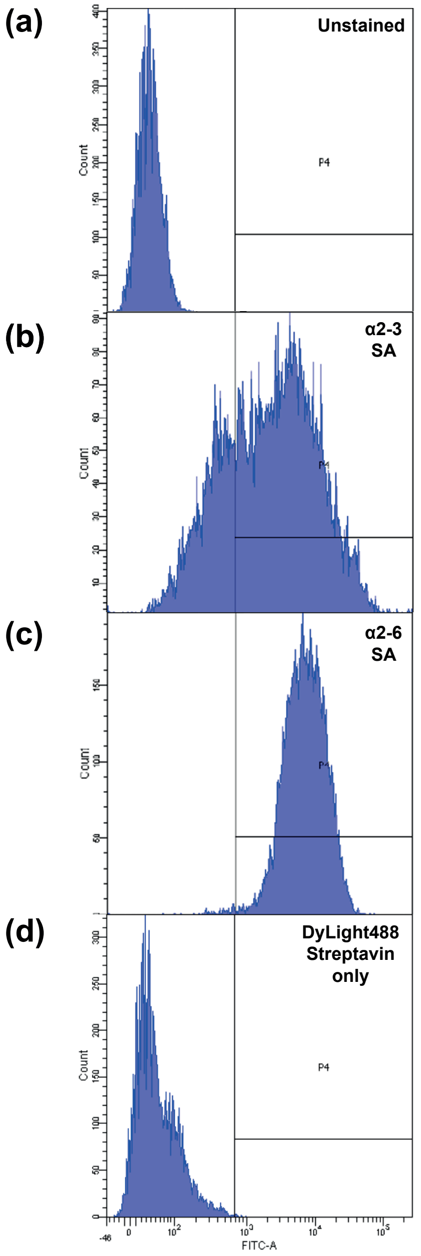

Figure S2. FACS analysis of α2-3- and α2-6-linked sialic acids on the surface of wdNHBE cells
NHBEs were differentiated at the air liquid interface for more than four weeks and cell surface α2-3- and α2-6-linked sialic acids bound by biotinylated *Maackia amurensis* agglutinin II (*MAA* II) and *Sambucus nigra* agglutinin I (*SNA* I) respectively were determined by FACS. Agglutinins were bound to DyLight 488 streptavidin, sorted and analysed. (**a**) Unstained wdNHBE cells were used as a negative control. (**b**) Cell-surface α2-3-linked sialic acids detected by *MAA* II were present on an average of 73.6% of wdNHBEs. (**c**) In contrast, 99.2% of airlifted NHBEs had α2-6-linked sialic acids on the cell surface as detected by *SNA* I binding. As for unstained wdNHBEs, (**d**) the DyLight 488 streptavidin-only treated cells indicated that there was no non-specific streptavidin binding.

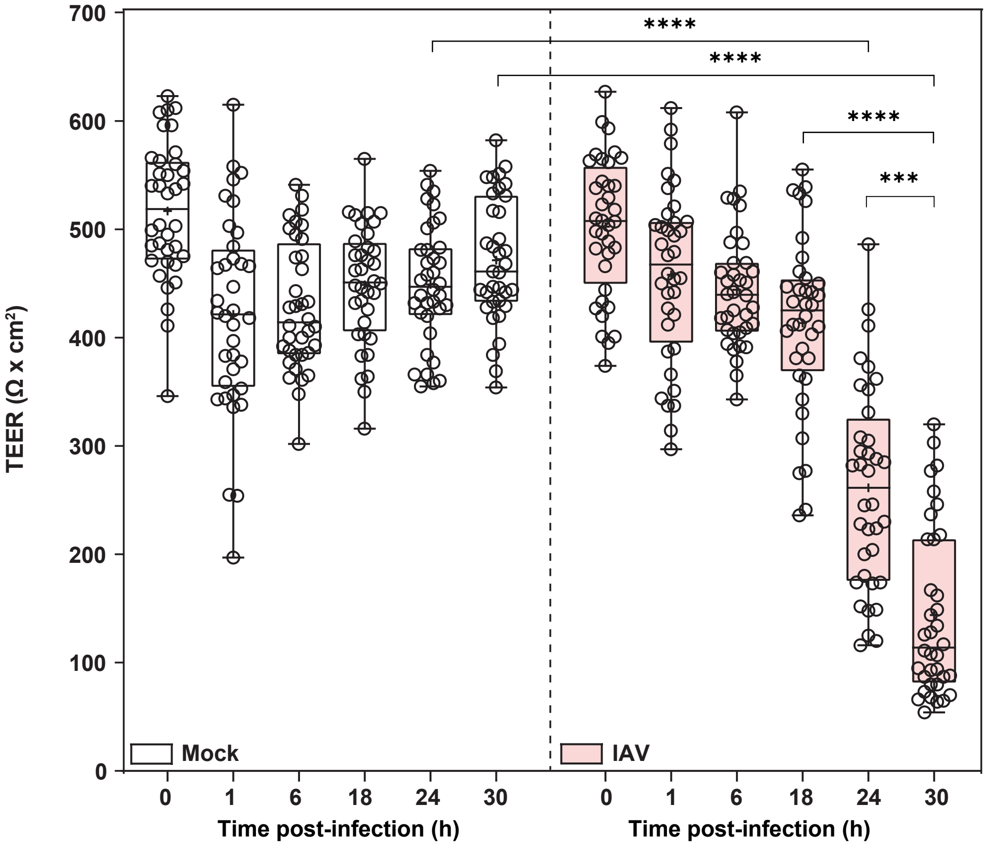

Figure S3. Transepithelial electrical resistance (TEER) readings for three independent experiments of Mock vs IAV H1N1pdm09 inoculated wdNHBE cells

wdNHBEs were inoculated with H1N1pdm09 (MO1 1) and barrier integrity (TEER, Ω x cm^2) compared to mock-inoculated cells pre-infection and 1, 6, 18, 24 and 30 h post-infection. At each timepoint, n=36 biological replicates for IAV and mock treatments were measured, apart from the 30 h mock treatment, n=35. Data are presented as box and whisker plots which show the mean (+), median, interquartile range and maximum and minimum values. The ends of each box represent the upper and lower quartiles, the horizontal line within the box indicates the median value and error bars show the minimum and maximum values. Individual values are depicted by open circles. Statistical significance is indicated: ***, p<0.001; ****, p<0.0001.

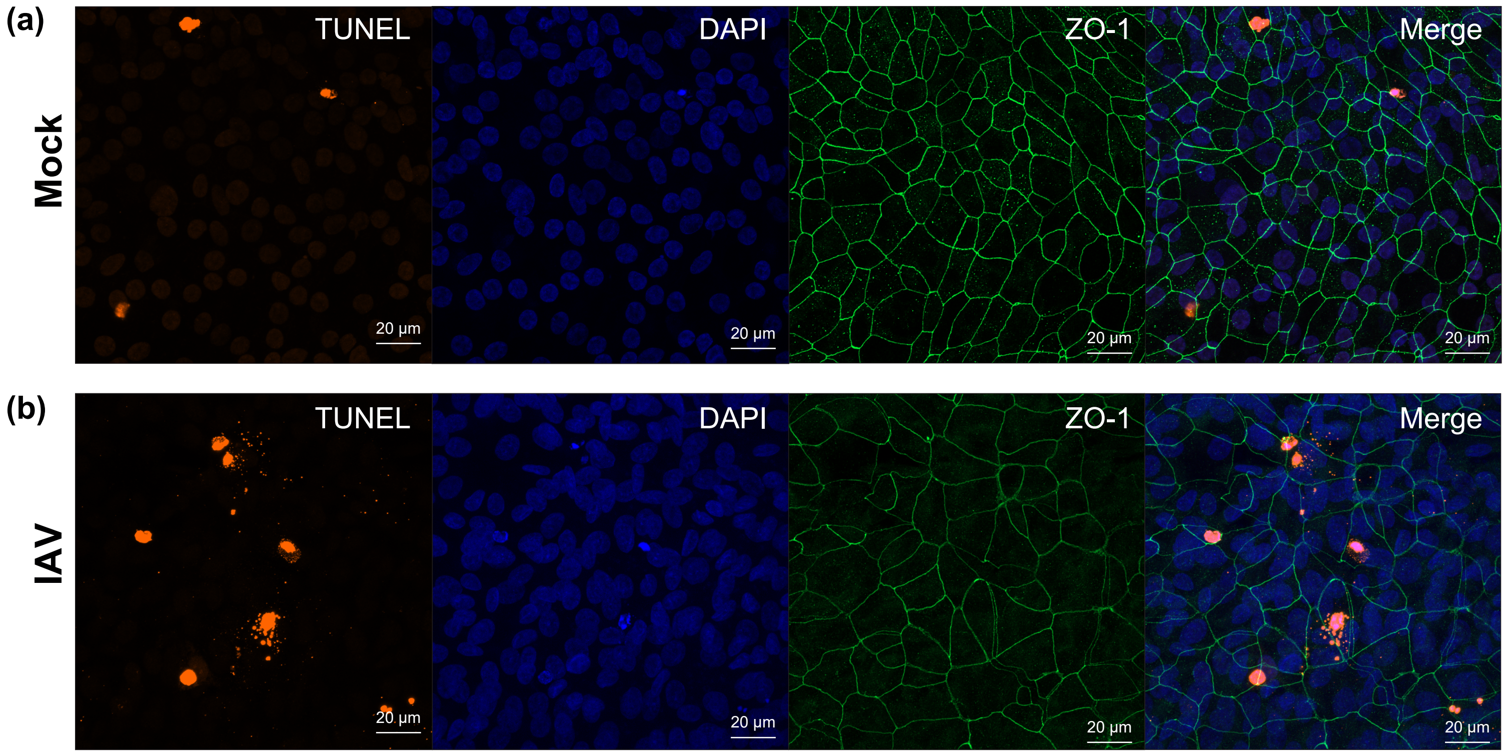

Figure S4. Apoptosis in wdNHBE cells infected with pandemic IAV H1N1pdm09
Airlifted NHBEs were inoculated with (**a**) ALI media alone (Mock) or with (**b**) IAV H1N1pdm09 added to the apical surface of cells differentiated on transwells. At 30 h post-infection, cells were fixed with 4% PFA, and TUNEL stained (orange, apoptotic cells), ZO-1 (green, tight junction protein 1, perijunctional belt) and DAPI (nuclei, blue). TUNEL staining shows the increased apoptosis characterised by blebbing and cell fragmentation in (**b**) IAV-infected versus (**a**) mock-inoculated cells. DAPI nuclear staining also shows the breakdown of cell nuclei undergoing programmed cell death, while ZO-1 staining suggests that IAV-iinfected cells are distended compared to mock-treated cells. Merge of TUNEL, ZO-1 and DAPI for (**a**) mock and (**b**) H1N1pdm09-infected cells. Maximum intensity projections of *en face* Z-stacks, 21 slices (10 µm) captured by confocal microscopy using a 40x oil immersion objective. A 20 µm scale bar indicates cell size.

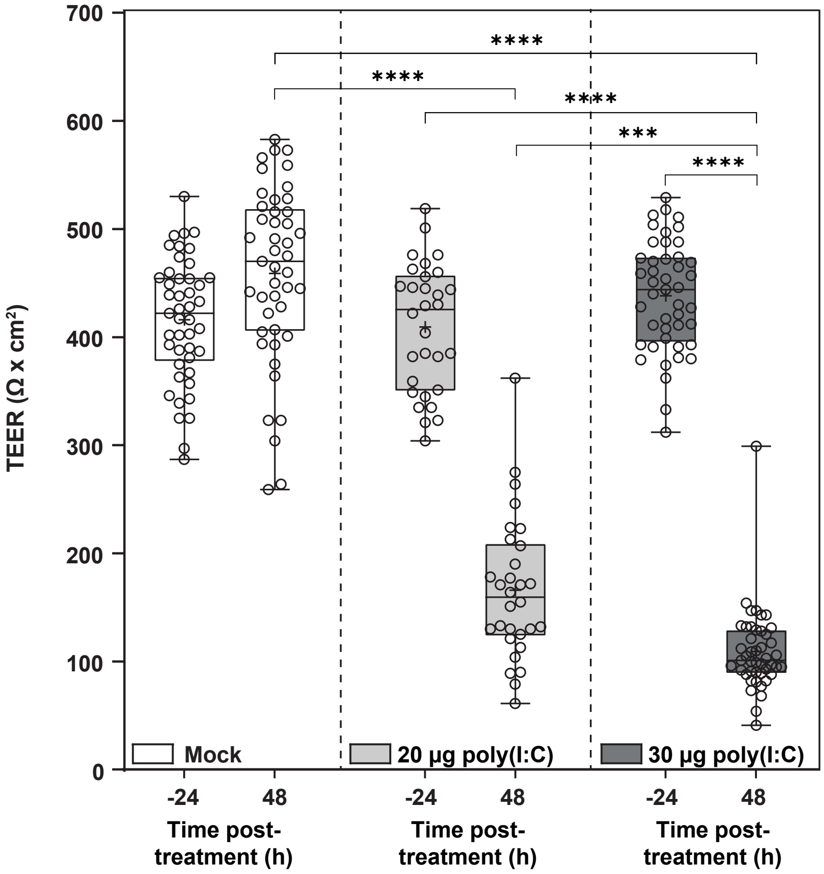

Figure S5. Transepithelial electrical resistance (TEER) readings for three independent experiments of wdNHBE cells treated with 20 ug & 30 ug poly(I:C) vs mock-treated cells

wdNHBEs treated with (20 µg or 30 µg) poly(I:C) for 24 h were assayed 48 h post-treatment and barrier integrity (TEER, Ω x cm^2) compared to mock (ALI media)-treated cells. For each timepoint n=45 biological replicates for mock; n=30 for 20 µg poly(I:C) and n=45 replicates for 30 µg poly(I:C). Data are presented as box and whisker plots which show the mean (+), median, interquartile range and maximum and minimum values. The ends of each box represent the upper and lower quartiles, the horizontal line within the box indicates the median value and error bars show the minimum and maximum values. Individual values are depicted by open circles. Statistical significance is indicated: ***, p<0.001; ****, p<0.0001.

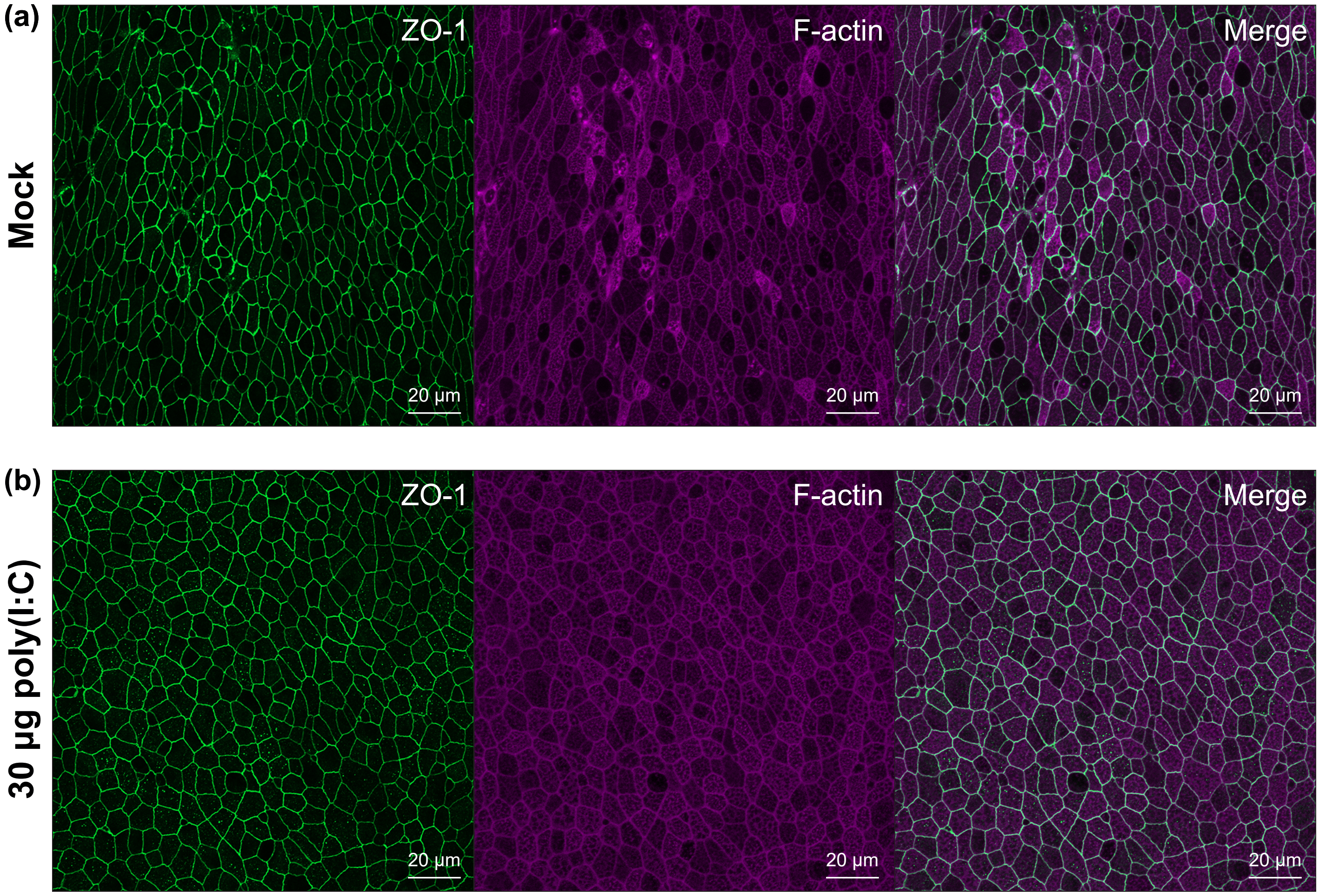

Figure S6. Immunofluorescence assays of poly(I:C)-treated wdNHBE cells
Airlifted NHBEs were treated with (**a**) ALI media alone (Mock) or (**b**) 30 µg poly(I:C) diluted in ALI media added to the apical surface of cells differentiated on transwells. After a 24 h incubation, the supernatant was removed from the apical surface and the cells incubated at the air liquid interface for a further 48 h at 37°C/5% CO_2. Representative images of cells fixed with 4% PFA and stained with ZO-1 (green) and phalloidin (F-actin, pink) are shown. ZO-1 formed an intact perijunctional belt around each cell in both the (**a**) Mock and (**b**) poly(I:C)-treated cells. Similarly, the cytoskeleton (F-actin filaments) appeared intact in both the mock and poly(I:C)-treated cells. Merge of ZO-1 and F-actin for (**a**) mock and (**b**) 30 µg poly(I:C)-treated cells. MAximum intensity projections of *en face* Z-stacks, 22 slices (10.5 µm) imaged by confocal microscopy using a 40x oil immersion objective. A 20 µm scale bar indicates cell size.

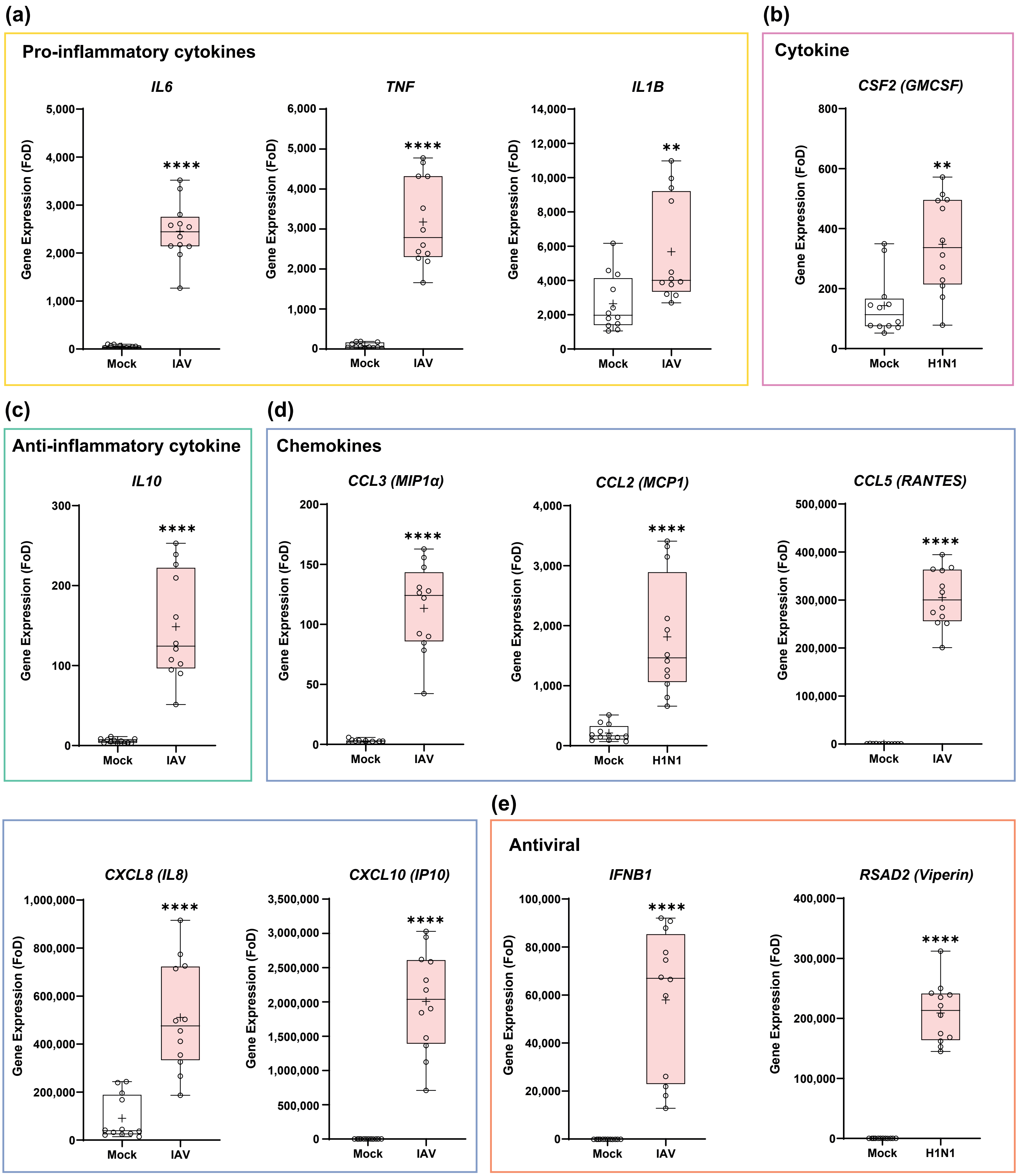

Figure S7. **Expression of cytokines, chemokines & antiviral genes in wdNHBE cells in response to pandemic influenza infection.** Gene expression in mock vs IAV H1N1pdm09-inoculated cells was determined 30 h post-inoculation by RT-qPCR. Expression was normalized to GAPDH and expressed as fold over detectable (FoD), with a minimum level detectable of 40 cycles (a) Pro-inflammatory cytokines: *IL6*, *TNF*, *IL1B*; (b) Cytokine: *CSF2* (*GMCSF*); (c) Anti-inflammatory cytokine: *IL10*; (d) Chemokines: *CCL3* (*MIP1α*), *CCL2* (*MCP1*), *CCL5* (*RANTES*), *CXCL8* (*IL8*), *CXCL10* (*IP10*); (e) Antivirals: *IFNB1*, *RSAD2 (viperin)*. Data from three independent experiments was pooled, analyzed by unpaired t-tests and expressed as mean ± SEM. Statistical significance of the expression of each gene for Mock (n=12) versus IAV-infected cells (n=12) was determined by unpaired t-tests. ns, not significant; **, p<0.01; ****, p<0.0001.

Table S4: Gene copy numbers for IAV H1N1pdm09 vs mock-infected wdNHBE cells

Expt.	Treat.	Well no.	Gene	Copy No.
a	Mock	1	*MUC5AC*	634.65
a	Mock	2	*MUC5AC*	291.93
a	Mock	3	*MUC5AC*	765.92
a	Mock	4	*MUC5AC*	2892.23
a	IAV	5	*MUC5AC*	1244.69
a	IAV	6	*MUC5AC*	911.98
a	IAV	7	*MUC5AC*	1043.07
a	IAV	8	*MUC5AC*	1147.75
b	Mock	1	*MUC5AC*	1649.40
b	Mock	2	*MUC5AC*	689.80
b	Mock	3	*MUC5AC*	3209.18
b	Mock	4	*MUC5AC*	1765.19
b	IAV	5	*MUC5AC*	1513.92
b	IAV	6	*MUC5AC*	1635.22
b	IAV	7	*MUC5AC*	2140.90
b	IAV	8	*MUC5AC*	2011.30
c	Mock	1	*MUC5AC*	1567.64
c	Mock	2	*MUC5AC*	1081.53
c	Mock	3	*MUC5AC*	2144.13
c	Mock	4	*MUC5AC*	1949.28
c	IAV	5	*MUC5AC*	1035.72
c	IAV	6	*MUC5AC*	4695.41
c	IAV	7	*MUC5AC*	1375.14
c	IAV	8	*MUC5AC*	1698.30
a	Mock	1	*MUC5B*	3204.94
a	Mock	2	*MUC5B*	4618.26
a	Mock	3	*MUC5B*	6706.97
a	Mock	4	*MUC5B*	5663.69
a	IAV	5	*MUC5B*	2123.13
a	IAV	6	*MUC5B*	2389.81
a	IAV	7	*MUC5B*	1997.59
a	IAV	8	*MUC5B*	1583.81
b	Mock	1	*MUC5B*	8018.78
b	Mock	2	*MUC5B*	7674.82
b	Mock	3	*MUC5B*	5815.49
b	Mock	4	*MUC5B*	6981.69
b	IAV	5	*MUC5B*	2710.46
b	IAV	6	*MUC5B*	2664.03
b	IAV	7	*MUC5B*	3022.77
b	IAV	8	*MUC5B*	3774.35
c	Mock	1	*MUC5B*	10684.24
c	Mock	2	*MUC5B*	9086.60
c	Mock	3	*MUC5B*	18973.43
c	Mock	4	*MUC5B*	10880.09
c	IAV	5	*MUC5B*	3488.19
c	IAV	6	*MUC5B*	14811.39
c	IAV	7	*MUC5B*	5734.23
c	IAV	8	*MUC5B*	5770.46

Expt.	Treat.	Well no.	Gene	Copy No.
a	Mock	1	*CCL5*	427.77
a	Mock	2	*CCL5*	416.07
a	Mock	3	*CCL5*	255.78
a	Mock	4	*CCL5*	545.37
a	IAV	5	*CCL5*	249786.26
a	IAV	6	*CCL5*	267323.80
a	IAV	7	*CCL5*	285345.39
a	IAV	8	*CCL5*	202287.31
b	Mock	1	*CCL5*	379.02
b	Mock	2	*CCL5*	1098.89
b	Mock	3	*CCL5*	318.67
b	Mock	4	*CCL5*	894.99
b	IAV	5	*CCL5*	329619.82
b	IAV	6	*CCL5*	365539.40
b	IAV	7	*CCL5*	397136.79
b	IAV	8	*CCL5*	318879.40
c	Mock	1	*CCL5*	113.93
c	Mock	2	*CCL5*	579.43
c	Mock	3	*CCL5*	98.25
c	Mock	4	*CCL5*	412.79
c	IAV	5	*CCL5*	363748.94
c	IAV	6	*CCL5*	370257.65
c	IAV	7	*CCL5*	254617.69
c	IAV	8	*CCL5*	276012.62
a	Mock	1	*IL6*	5.59
a	Mock	2	*IL6*	16.13
a	Mock	3	*IL6*	21.10
a	Mock	4	*IL6*	48.84
a	IAV	5	*IL6*	2142.42
a	IAV	6	*IL6*	2173.26
a	IAV	7	*IL6*	1975.68
a	IAV	8	*IL6*	1271.41
b	Mock	1	*IL6*	71.65
b	Mock	2	*IL6*	98.73
b	Mock	3	*IL6*	96.87
b	Mock	4	*IL6*	54.20
b	IAV	5	*IL6*	2616.05
b	IAV	6	*IL6*	3533.47
b	IAV	7	*IL6*	3356.17
b	IAV	8	*IL6*	2585.64
c	Mock	1	*IL6*	27.37
c	Mock	2	*IL6*	8.92
c	Mock	3	*IL6*	73.76
c	Mock	4	*IL6*	24.95
c	IAV	5	*IL6*	2547.39
c	IAV	6	*IL6*	2811.38
c	IAV	7	*IL6*	2142.24
c	IAV	8	*IL6*	2349.91

Expt.	Treat.	Well no.	Gene	Copy No.
a	Mock	1	*IL10*	3.39
a	Mock	2	*IL10*	3.25
a	Mock	3	*IL10*	3.82
a	Mock	4	*IL10*	2.58
a	IAV	5	*IL10*	102.33
a	IAV	6	*IL10*	90.23
a	IAV	7	*IL10*	94.90
a	IAV	8	*IL10*	51.33
b	Mock	1	*IL10*	5.72
b	Mock	2	*IL10*	11.23
b	Mock	3	*IL10*	7.14
b	Mock	4	*IL10*	8.46
b	IAV	5	*IL10*	238.85
b	IAV	6	*IL10*	210.29
b	IAV	7	*IL10*	252.88
b	IAV	8	*IL10*	160.97
c	Mock	1	*IL10*	8.43
c	Mock	2	*IL10*	4.92
c	Mock	3	*IL10*	3.07
c	Mock	4	*IL10*	6.41
c	IAV	5	*IL10*	120.89
c	IAV	6	*IL10*	226.95
c	IAV	7	*IL10*	106.98
c	IAV	8	*IL10*	127.57
a	Mock	1	*IL1B*	2396.13
a	Mock	2	*IL1B*	2093.43
a	Mock	3	*IL1B*	1791.30
a	Mock	4	*IL1B*	1862.47
a	IAV	5	*IL1B*	4507.22
a	IAV	6	*IL1B*	3927.04
a	IAV	7	*IL1B*	3780.67
a	IAV	8	*IL1B*	3139.78
b	Mock	1	*IL1B*	3491.57
b	Mock	2	*IL1B*	4585.17
b	Mock	3	*IL1B*	6192.51
b	Mock	4	*IL1B*	4374.14
b	IAV	5	*IL1B*	10983.26
b	IAV	6	*IL1B*	8673.16
b	IAV	7	*IL1B*	9938.89
b	IAV	8	*IL1B*	9435.43
c	Mock	1	*IL1B*	1127.20
c	Mock	2	*IL1B*	1474.32
c	Mock	3	*IL1B*	1372.69
c	Mock	4	*IL1B*	1056.06
c	IAV	5	*IL1B*	2705.35
c	IAV	6	*IL1B*	3883.29
c	IAV	7	*IL1B*	3219.25
c	IAV	8	*IL1B*	4109.90

Expt.	Treat.	Well no.	Gene	Copy No.
a	Mock	1	*IFNB1*	11.88
a	Mock	2	*IFNB1*	9.72
a	Mock	3	*IFNB1*	22.54
a	Mock	4	*IFNB1*	11.92
a	IAV	5	*IFNB1*	91071.38
a	IAV	6	*IFNB1*	67807.74
a	IAV	7	*IFNB1*	88319.09
a	IAV	8	*IFNB1*	78070.26
b	Mock	1	*IFNB1*	14.49
b	Mock	2	*IFNB1*	7.84
b	Mock	3	*IFNB1*	14.48
b	Mock	4	*IFNB1*	17.12
b	IAV	5	*IFNB1*	92572.61
b	IAV	6	*IFNB1*	74904.71
b	IAV	7	*IFNB1*	59998.07
b	IAV	8	*IFNB1*	66825.58
c	Mock	1	*IFNB1*	4.34
c	Mock	2	*IFNB1*	1.88
c	Mock	3	*IFNB1*	6.52
c	Mock	4	*IFNB1*	3.14
c	IAV	5	*IFNB1*	18144.24
c	IAV	6	*IFNB1*	21976.53
c	IAV	7	*IFNB1*	26123.08
c	IAV	8	*IFNB1*	12880.97
a	Mock	1	*RSAD2*	524.93
a	Mock	2	*RSAD2*	592.33
a	Mock	3	*RSAD2*	569.64
a	Mock	4	*RSAD2*	428.39
a	IAV	5	*RSAD2*	236792.01
a	IAV	6	*RSAD2*	222712.12
a	IAV	7	*RSAD2*	240972.81
a	IAV	8	*RSAD2*	251944.33
b	Mock	1	*RSAD2*	479.45
b	Mock	2	*RSAD2*	506.54
b	Mock	3	*RSAD2*	574.17
b	Mock	4	*RSAD2*	530.22
b	IAV	5	*RSAD2*	175875.72
b	IAV	6	*RSAD2*	206723.25
b	IAV	7	*RSAD2*	145822.37
b	IAV	8	*RSAD2*	153849.27
c	Mock	1	*RSAD2*	471.38
c	Mock	2	*RSAD2*	873.51
c	Mock	3	*RSAD2*	807.85
c	Mock	4	*RSAD2*	344.99
c	IAV	5	*RSAD2*	163328.50
c	IAV	6	*RSAD2*	243507.79
c	IAV	7	*RSAD2*	314038.25
c	IAV	8	*RSAD2*	169494.31

Expt.	Treat.	Well no.	Gene	Copy No.
a	Mock	1	*CXCL8*	14129.17
a	Mock	2	*CXCL8*	21003.84
a	Mock	3	*CXCL8*	26975.43
a	Mock	4	*CXCL8*	23825.00
a	IAV	5	*CXCL8*	327128.53
a	IAV	6	*CXCL8*	268193.68
a	IAV	7	*CXCL8*	356166.99
a	IAV	8	*CXCL8*	187140.43
b	Mock	1	*CXCL8*	169189.81
b	Mock	2	*CXCL8*	240617.22
b	Mock	3	*CXCL8*	196054.36
b	Mock	4	*CXCL8*	245103.26
b	IAV	5	*CXCL8*	921104.99
b	IAV	6	*CXCL8*	718278.51
b	IAV	7	*CXCL8*	779639.21
b	IAV	8	*CXCL8*	730537.26
c	Mock	1	*CXCL8*	37066.72
c	Mock	2	*CXCL8*	32507.44
c	Mock	3	*CXCL8*	44707.62
c	Mock	4	*CXCL8*	41618.12
c	IAV	5	*CXCL8*	506186.89
c	IAV	6	*CXCL8*	457833.51
c	IAV	7	*CXCL8*	414265.02
c	IAV	8	*CXCL8*	500806.58
a	Mock	1	*CSF2*	70.31
a	Mock	2	*CSF2*	70.37
a	Mock	3	*CSF2*	125.36
a	Mock	4	*CSF2*	136.54
a	IAV	5	*CSF2*	209.09
a	IAV	6	*CSF2*	171.86
a	IAV	7	*CSF2*	228.78
a	IAV	8	*CSF2*	78.52
b	Mock	1	*CSF2*	147.45
b	Mock	2	*CSF2*	350.70
b	Mock	3	*CSF2*	172.94
b	Mock	4	*CSF2*	328.35
b	IAV	5	*CSF2*	573.40
b	IAV	6	*CSF2*	515.51
b	IAV	7	*CSF2*	465.89
b	IAV	8	*CSF2*	492.85
c	Mock	1	*CSF2*	51.64
c	Mock	2	*CSF2*	91.71
c	Mock	3	*CSF2*	95.04
c	Mock	4	*CSF2*	88.70
c	IAV	5	*CSF2*	495.57
c	IAV	6	*CSF2*	362.00
c	IAV	7	*CSF2*	272.45
c	IAV	8	*CSF2*	312.82

Expt.	Treat.	Well no.	Gene	Copy No.
a	Mock	1	*CXCL10*	59.92
a	Mock	2	*CXCL10*	77.50
a	Mock	3	*CXCL10*	57.41
a	Mock	4	*CXCL10*	95.63
a	IAV	5	*CXCL10*	1368903.21
a	IAV	6	*CXCL10*	1131700.42
a	IAV	7	*CXCL10*	1486557.38
a	IAV	8	*CXCL10*	714472.46
b	Mock	1	*CXCL10*	270.53
b	Mock	2	*CXCL10*	453.13
b	Mock	3	*CXCL10*	321.04
b	Mock	4	*CXCL10*	178.72
b	IAV	5	*CXCL10*	2967048.18
b	IAV	6	*CXCL10*	2603871.08
b	IAV	7	*CXCL10*	3055053.89
b	IAV	8	*CXCL10*	2640456.49
c	Mock	1	*CXCL10*	49.12
c	Mock	2	*CXCL10*	64.60
c	Mock	3	*CXCL10*	69.86
c	Mock	4	*CXCL10*	120.09
c	IAV	5	*CXCL10*	2191444.98
c	IAV	6	*CXCL10*	2335840.71
c	IAV	7	*CXCL10*	1916335.90
c	IAV	8	*CXCL10*	1858520.77
a	Mock	1	*CCL3*	5.79
a	Mock	2	*CCL3*	
a	Mock	3	*CCL3*	3.39
a	Mock	4	*CCL3*	2.46
a	IAV	5	*CCL3*	89.77
a	IAV	6	*CCL3*	78.44
a	IAV	7	*CCL3*	127.68
a	IAV	8	*CCL3*	42.06
b	Mock	1	*CCL3*	2.60
b	Mock	2	*CCL3*	
b	Mock	3	*CCL3*	
b	Mock	4	*CCL3*	3.69
b	IAV	5	*CCL3*	155.39
b	IAV	6	*CCL3*	125.94
b	IAV	7	*CCL3*	130.66
b	IAV	8	*CCL3*	122.15
c	Mock	1	*CCL3*	
c	Mock	2	*CCL3*	3.03
c	Mock	3	*CCL3*	2.74
c	Mock	4	*CCL3*	2.90
c	IAV	5	*CCL3*	148.02
c	IAV	6	*CCL3*	160.05
c	IAV	7	*CCL3*	91.78
c	IAV	8	*CCL3*	83.60

Expt.	Treat.	Well no.	Gene	Copy No.
a	Mock	1	*CCL2*	87.58
a	Mock	2	*CCL2*	180.34
a	Mock	3	*CCL2*	138.28
a	Mock	4	*CCL2*	170.70
a	IAV	5	*CCL2*	806.32
a	IAV	6	*CCL2*	1417.10
a	IAV	7	*CCL2*	1162.54
a	IAV	8	*CCL2*	661.78
b	Mock	1	*CCL2*	238.10
b	Mock	2	*CCL2*	513.77
b	Mock	3	*CCL2*	358.13
b	Mock	4	*CCL2*	391.87
b	IAV	5	*CCL2*	3327.28
b	IAV	6	*CCL2*	1521.17
b	IAV	7	*CCL2*	3421.91
b	IAV	8	*CCL2*	2129.87
c	Mock	1	*CCL2*	69.54
c	Mock	2	*CCL2*	92.23
c	Mock	3	*CCL2*	145.96
c	Mock	4	*CCL2*	158.16
c	IAV	5	*CCL2*	1033.03
c	IAV	6	*CCL2*	3160.51
c	IAV	7	*CCL2*	1936.95
c	IAV	8	*CCL2*	1265.67
a	Mock	1	*TNF*	32.42
a	Mock	2	*TNF*	42.98
a	Mock	3	*TNF*	17.70
a	Mock	4	*TNF*	49.72
a	IAV	5	*TNF*	2991.31
a	IAV	6	*TNF*	2608.89
a	IAV	7	*TNF*	2396.59
a	IAV	8	*TNF*	1665.97
b	Mock	1	*TNF*	129.88
b	Mock	2	*TNF*	187.89
b	Mock	3	*TNF*	173.85
b	Mock	4	*TNF*	189.73
b	IAV	5	*TNF*	4797.87
b	IAV	6	*TNF*	4682.70
b	IAV	7	*TNF*	4332.55
b	IAV	8	*TNF*	4323.47
c	Mock	1	*TNF*	16.60
c	Mock	2	*TNF*	49.06
c	Mock	3	*TNF*	65.91
c	Mock	4	*TNF*	40.87
c	IAV	5	*TNF*	2202.15
c	IAV	6	*TNF*	3535.36
c	IAV	7	*TNF*	2279.62
c	IAV	8	*TNF*	2443.89

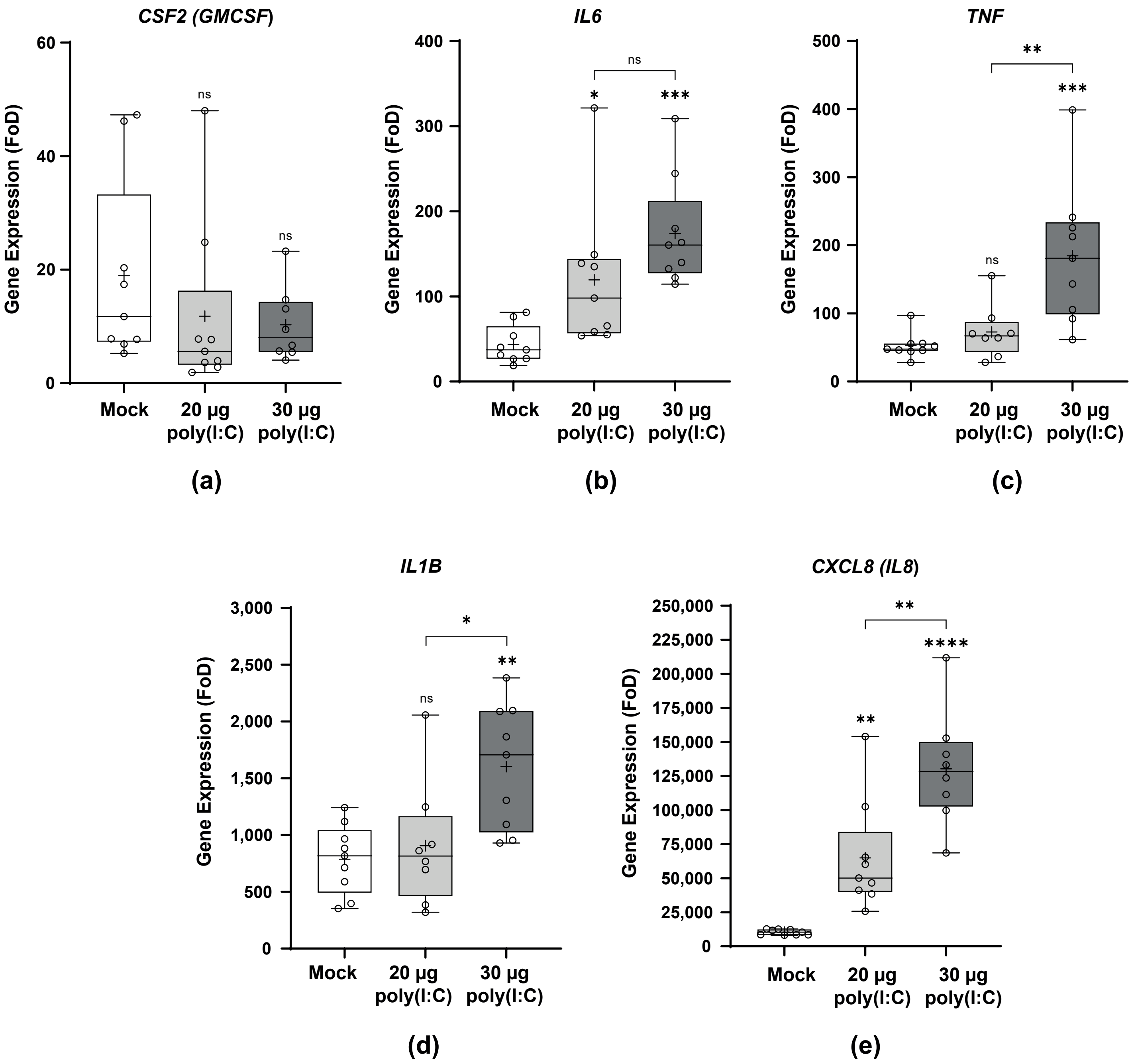

Figure S8. Poly(I:C) stimulation of innate immune response genes in wdNHBE cells

Gene expression in wdNHBEs 48 h post-treatment with 20 µg and 30 µg poly(I:C) was assayed by RT-qPCR and compared to mock (ALI media)-treated cells. Gene expression is shown as fold over detectable (FoD), where the minimum level detectable was set at 40 cycles. Expression was normalized to *GAPDH* and data pooled from three independent experiments. (**a**) *CSF2* (*GMCSF*), (**b**) *IL6*, (**c**) *TNF*, (**d**) *IL1B* and (**e**) *CXCL8* (*IL8)*. Data are presented as box and whisker plots which show the mean (+), median, interquartile range and maximum and minimum values. The ends of each box represent the upper and lower quartiles, the horizontal line within the box shows the median value and error bars indicate the minimum and maximum values. Individual values are depicted by open circles. The statistical significance of poly(I:C) versus mock treated cells was determined by one-way ANOVA using Tukey's multiple comparison test. ns, not significant; *, p<0.05; **, p<0.01; ***, p<0.001; ****, p<0.0001. The number of biological replicates per gene and per treatment are mock, n=9 per gene; 20 µg poly(I:C), n=9 per gene (n=8 for *IL1B* and *TNF*) and 30 µg poly(I:C), n=9 per gene (n=8 for *CXCL8*).

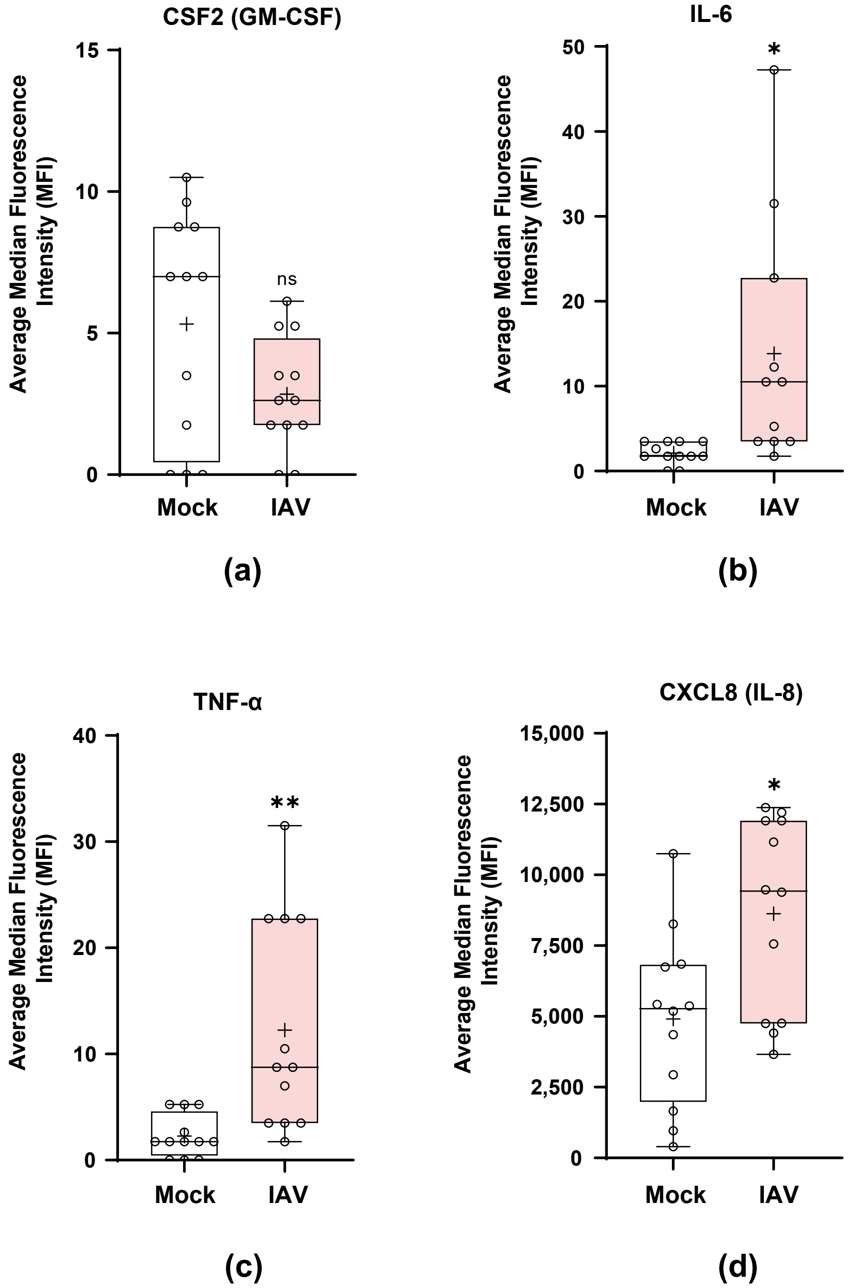

Figure S9. The basolateral secretion of cytokines and chemokines by wdNHBE cells in response to infection with IAV H1N1pdm09

The proinflammatory cytokines CSF2, IL-6 and TNF-α and the CXCL8 chemokine secreted into the basolateral (lower) compartment by wdNHBE cells infected with H1N1pdm09 or mock-inoculated with ALI media were assayed 30 h post-infection by multiplex immunoassays. At 24 h post-IAV infection, basolateral media was replaced and then collected 6 h later at the experiment endpoint. Data from three independents was pooled and analysed by unpaired two-tailed t-tests. Data are presented as the average median fluorescence intensity (MFI) of n=12 biological replicates for GM-CSF, TNF-α and CXCL8 and n=11 replicates for IL-6. IL-1ß was omitted as values were at the minimum level of detection. Data are depicted as box and whisker plots which show the mean (+), median, interquartile range and maximum and minimum values. The ends of each box represent the upper and lower quartiles, the horizontal line within the box shows the median value and error bars indicate the minimum and maximum values. Individual values are depicted by open circles. Statistical significance is indicated: ns, not significant; *, $p<0.05$; **, $p<0.01$.

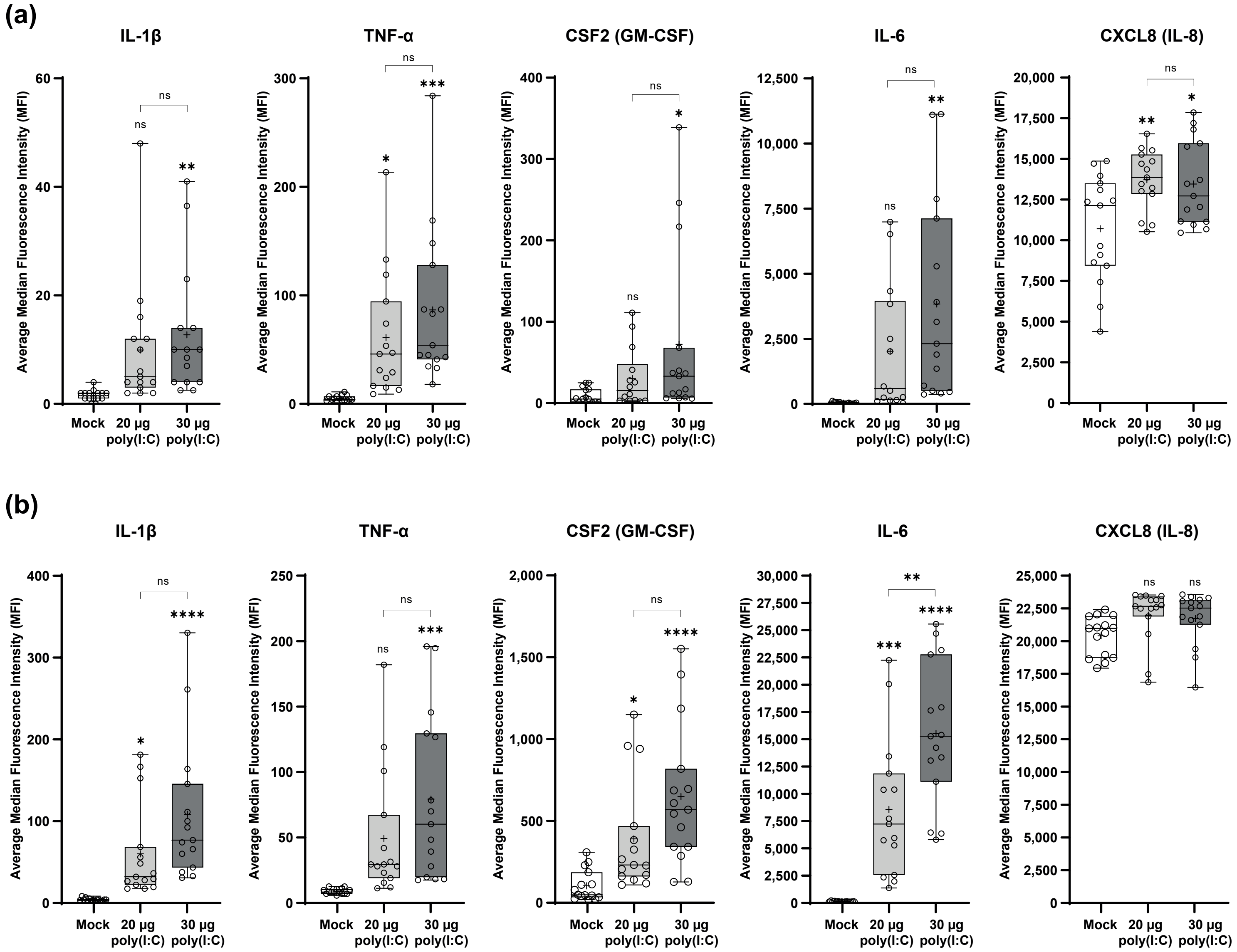

Figure S10. The apical and basolateral secretion of cytokines and chemokines by wdNHBE cells in response to poly(I:C) treatment

The proinflammatory cytokines IL-1β, CSF2, TNF-α and IL-6, and the CXCL8 chemokine secreted into the **(a)** apical and **(b)** basolateral compartments by wdNHBE cells were assayed 48 h post-treatment with 20 and 30 µg poly(I:C) and compared to mock-treated (ALI media) cells by multiplex immunoassays. Apical samples were collected at the experiment endpoint. ALI media was added to the apical surface, the cells incubated for 30 min and apical proteins secreted into the supernatant collected. Basolateral media was changed 24 h post-poly(I:C) innoculation and then collected 48 h later on Day 3. Data from three independent experiments was combined and analysed by one-way ANOVA using Tukey's multiple comparison test. Data are presented as box and whisker plots which show the mean (+), median, interquartile range and maximum and minimum values. The ends of each box represent the upper and lower quartiles, the horizontal line within the box indicates the median value and error bars show the minimum and maximum values. Individual values are depicted by open circles. n=45 biological replicates per cytokine, 15 per treatment were analysed for IL-1β, TNF-α, CSF2, IL-6, and CXCL8. Statistical significance is indicated: ns, not significant; *, $p<0.05$; **, $p<0.01$; ***, $p<0.001$; ****, $P<0.0001$.
